# Mapping Plastic and Plastic Additive Cycles in Coastal Countries: A Norwegian Case Study

**DOI:** 10.1021/acs.est.3c09176

**Published:** 2024-05-04

**Authors:** Ahmed Marhoon, Miguel Las Heras Hernandez, Romain Guillaume Billy, Daniel Beat Müller, Francesca Verones

**Affiliations:** † Industrial Ecology Programme, Department of Energy and Process Engineering, Norwegian University of Science and Technology (NTNU), Trondheim NO-7034, Norway; ‡ The Climate and Environmental Research Institute (NILU), Trondheim NO-7013, Norway

**Keywords:** plastic pollution, plastic additives, plastic cycle, marine plastic pollution, material flow analysis, combined lifetime-leaching

## Abstract

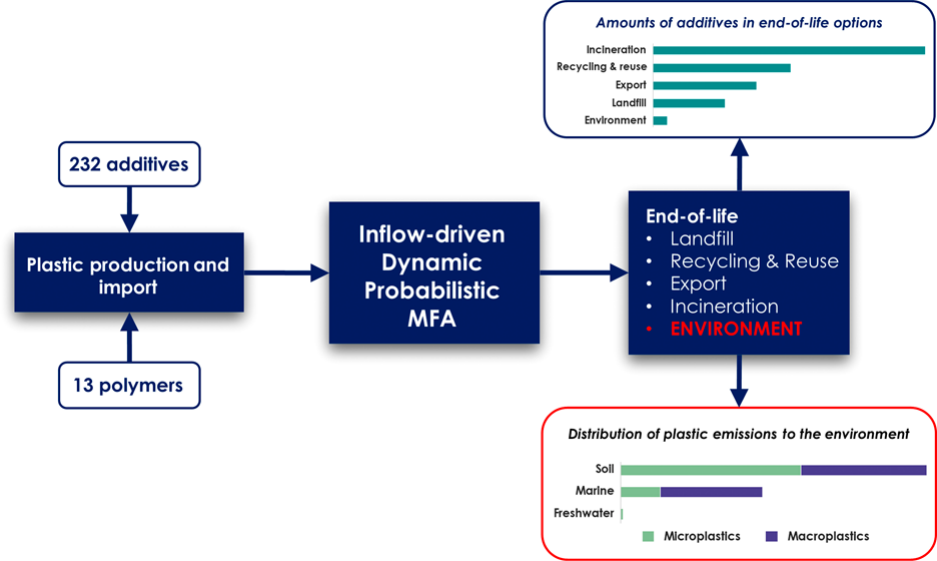

The growing environmental consequences caused by plastic pollution highlight the need for a better understanding of plastic polymer cycles and their associated additives. We present a novel, comprehensive top-down method using inflow-driven dynamic probabilistic material flow analysis (DPMFA) to map the plastic cycle in coastal countries. For the first time, we covered the progressive leaching of microplastics to the environment during the use phase of products and modeled the presence of 232 plastic additives. We applied this methodology to Norway and proposed initial release pathways to different environmental compartments. 758 kt of plastics distributed among 13 different polymers was introduced to the Norwegian economy in 2020, 4.4 Mt was present in in-use stocks, and 632 kt was wasted, of which 15.2 kt (2.4%) was released to the environment with a similar share of macro- and microplastics and 4.8 kt ended up in the ocean. Our study shows tire wear rubber as a highly pollutive microplastic source, while most macroplastics originated from consumer packaging with LDPE, PP, and PET as dominant polymers. Additionally, 75 kt of plastic additives was potentially released to the environment alongside these polymers. We emphasize that upstream measures, such as consumption reduction and changes in product design, would result in the most positive impact for limiting plastic pollution.

## Abstract 2

A quantification of the plastic and additive cycle for coastal countries is missing. This study presents a novel method to map plastic and plastic additive cycles and applies it to Norway as a case study.

## Introduction

Plastic as a material is versatile and cheap and thus satisfies a wide range of societal needs.^1^ Since ∼1950, plastic production has increased 230-fold,^2^ making plastics one of the most abundant human-made materials, and future trends do not show any slowdown.^3^ This ever-increasing growth coupled with poor waste management practices in many world regions has led to an unceasing release of plastics into the environment. Due to its persistency and slow degradation rate, reaching up to thousands of years,^4^ plastic has accumulated extensively, becoming pervasive and infiltrating even the most remote places, from Mt. Everest^5^ to the deepest parts of the oceans.^6^ At the moment, a majority of plastic items are derived from nonrenewable organic hydrocarbons (e.g., oil and gas). The basic building blocks (i.e., polymers) are very diverse, each with distinctive chemical and physical properties,^7,8^ but also portray a wide range of toxicity levels and behaviors. A large number of additives and processing aids,^9^ many of which are classified as hazardous,^10−14^ is being used during plastic production to enhance sought-after properties or functionality.^8,15^ These substances are often understudied or inadequately regulated.^8^

Understanding plastic polymer flows throughout their entire life cycle is the first step in assessing the environmental performance of the plastic economy and in finding solutions to mitigate its impacts. The diverse polymer composition, varying product lifetimes and applications, and different release mechanisms pose a challenge for estimating plastic losses to the environment. The literature generally distinguishes plastic pollution into microplastics (<5 mm) and macroplastics (>5 mm).^16^ Several studies provided a rough estimation of the overall release of microplastics^17,18^ and macroplastics,^19,20^ without distinguishing polymer types^18−21^ and using top-down,^18,19,22^ as well as bottom-up methods.^21^ Global models such as the one presented by Jambeck et al.^19^ are good to estimate the overall release of plastic to the environment at the global scale but fail to capture details regarding the pollution sources and polymer types. Distinguishing polymer types is essential when carrying out risk assessments for the studied products since different plastic polymers have various toxicity levels as well as different behaviors in the environmental compartments.

Material flow analysis (MFA) can provide a systemic view by mapping the flows and accumulation of plastic polymers used in different product applications in the economy and their release to the environment, further connecting the source of pollution to the final sinks. Previous studies used MFA to study the plastic cycle,^23−26^ for detailed polymers, product- and sector-specific assessments,^27,28^ including their flows to the environment,^29−32^ and using inflow-driven dynamic models^32−35^ to give a detailed picture of the current amounts remaining in in-use stocks and those found in sinks. However, the progressive release of microplastics from wear and tear processes is currently misrepresented in these assessments and usually modeled in the same way as lifetime-related outflows, resulting in the attribution of the overall releases throughout the entire lifetime of products to a single model time step (e.g., one year).^29,32^ It is essential to consider the stock dynamics governing such flows to accurately estimate the microplastic releases to the environment. Combined lifetime-leaching models have already been presented,^36,37^ but their use was limited to represent random destruction causes for products such as buildings and cars, and not to model wear and tear processes.

Differentiating initial releases to different environmental compartments relies heavily on the geographical location of the studied regions. Although the release of plastics to the marine environment has been covered in previous MFA models,^32^ these models failed to apply specific initial release pathways for coastal nations and therefore could have potentially led to an underestimation of the released plastic amounts to different environmental compartments, including the marine environment as the final sink.

MFA has also been used to analyze various plastic additives’ cycles, such as bisphenol A (BPA),^38^ di(2-ethylhexhyl) phthalate (DEHP),^39,40^ and polybrominated diphenyl ethers (PBDEs),^41,42^ or additives present in specific plastic polymers (e.g., PVC).^43,44^ These studies estimated their production volumes and in-use stocks, as well as their emissions to the environment. However, no study has yet presented a polymer- and sector-dependent as well as economy-wide assessment of additives present in the plastic cycle.

The aim of this study is to provide a comprehensive tool to estimate polymer- and additive-specific flows in the anthroposphere and to the environment for coastal countries, with a high product category resolution. We used a dynamic probabilistic material flow analysis (DPMFA) model to quantify the use, release, and accumulation of macro- and microplastics into different environmental compartments. We propose a novel combined lifetime-leaching approach to simultaneously model end-of-life and wear and tear releases. In addition, we estimated the potential presence of commonly used additives in plastic products. As a case study, we applied our model to Norway for 13 different polymer types and 232 additives. We then highlight the major sources of plastic pollution and provide recommendations to help pinpoint and prioritize relevant policy interventions.

## Methods

### System Definition

Our model consists of 184 processes in total, of which six are production and manufacturing processes, 10 plastic application sectors with 49 individual product categories, 14 waste collection processes, six recycling systems processes, five anthropogenic sinks, 10 environmental sinks, and 65 processes that describe the plastic release pathways to these environmental sinks. A detailed material flow model describing the relationship of the model processes is shown in Figures S1–S4 in Supporting Information 1 (SI1), while Section S2 contains more details regarding all system processes. The model covers 13 different plastic polymers (LDPE, HDPE, PP, PS, PET, EPS, PVC, PUR, PA, PC, ABS, cellulose acetate (CA), and rubber) that make up ∼87% of the European plastic demand,^45^ distributed into 10 plastic application sectors: packaging, building and construction (B&C), agriculture, automotive, electrical and electronic equipment (EEE), boats and fisheries (B&F), clothing, household textiles, technical textiles, and others. We cover product categories that are considered as durable applications with long expected lifetimes, as well as single-use plastics that are short-lived.

### Modeling Approach

The plastic cycle was modeled using inflow-driven DPMFA, which was introduced by Bornhöft et al.,^46^ and the reader is referred to this study for the detailed DPMFA theory. In inflow-driven dynamic models, the entire system dynamics is determined by the inflow of mass, transfer coefficients (TCs), and lifetime functions. The TCs define the partitioning and distribution of a good or substance for the mass leaving a process to the next.^47^ The lifetime functions describe the residing time (e.g., years) of a product in the stocks before it is released as an outflow. This results in a delayed release of outflows and a buildup of mass in stocks, which mimics the physical state of the economy. In this model, the import of finished and semifinished plastic materials as well as virgin and recycled materials acts as the mass flowing into the system. This inflow is coupled with predefined TCs and product-specific lifetime functions to determine the flows to the application sectors, which act as stocks, and further into subsequent processes until finally reaching the final sinks where an accumulation of mass takes place.

The uncertainties in DPMFA are modeled using the approach presented by Laner et al.,^48^ which allows for using data with varying qualities from a wide range of sources. Bayesian probability distributions for the inflows, TCs, and lifetime functions, based on coefficients of variation (CVs), are generated. CVs are determined with a pedigree matrix for five data quality indicators that allows translating qualitative information into quantitative data, namely, geographical, temporal, and material fit, as well as completeness and source reliability. We chose a triangular distribution to allow using multiple data sources when available, where a triangular distribution is built for one data point, a trapezoidal distribution for two data points, and a step distribution for more than two data points. For TCs, a truncation of the distribution is necessary at 0 and 1 to avoid unphysical distributions. We limit the construction of TC distributions to a trapezoidal form by taking only high and low values into account. The pedigree matrix used in this study can be found in Table S13 in SI1. The model is run 10,000 times in a Monte Carlo simulation where in each run, a sample is chosen from the Bayesian distributions for each inflow, TCs, and lifetime and subsequently the final mass in each compartment is quantified. The results then constitute the mean and standard deviation of the model processes based on the results of the Monte Carlo simulation.

### Plastic Release and Accumulation

We combined the general plastic release method introduced by Kawecki and Nowack^29^ and the approach presented by Sieber et al.^35^ for tire abrasion-related flows. Since these models were designed for Switzerland, being a land-locked country, we included the needed processes to model the release of plastics to the marine environment for coastal countries. We distinguished between the releases of macro- and microplastics. The environmental sinks considered in this study are ocean, ocean sediments, beaches, freshwater shorelines, freshwater sediments, agricultural soil, roadside soil, subsurface soil, residential soil, and natural soil (see Table S2 in SI1 for a detailed description of these compartments). We further applied transport and redistribution modeling in the aquatic compartments after the initial releases. Detailed plastic release and accumulation modeling approaches can be found in Section S3.2 in SI1.

### Combined Lifetime-Leaching Approach

Processes such as wear and tear, washing, drying, and shedding lead to friction forces that cause the release of microparticles from different plastic products to different compartments (Table 1). A correction factor was applied to the lifetime-dependent outflows and stocks to account for the leaching of these product categories. Details on the combined lifetime-leaching methodology can be found in Section S5 in SI1.

**Table 1:** Summary of Product Categories That Are Relevant for the Leaching of Microplastics, the Responsible Leaching Process, and the Receiving Primary Compartment

**sector**	**product category**	**leaching mechanism**	**receiving compartment**
B&C	wall and floor coverings	wear and tear	indoor air
	pipes and ducts	wear and tear	residential soil, subsurface soil
	geotextiles	wear and tear	subsurface soil
agriculture	agricultural films	wear and tear	agricultural soil
	agricultural pipes	wear and tear	agricultural soil
	other agricultural plastics	wear and tear	agricultural soil
	agrotextiles	wear and tear	agricultural soil
other	fabric coatings	wear and tear	residential soil, natural soil, wastewater, stormwater
	household plastics	wear and tear	indoor air
clothing	clothing	washing, drying, and wear and tear	indoor air, outdoor air, wastewater, mixed waste collection
	technical clothing		
household textiles	household textiles		
	technical household textiles		
automotive	tires	wear due to friction forces with road surfaces	outdoor air, highways, other roads

### Plastic Additives

An estimation of the maximum potential quantities of additives contained in plastic products was calculated by coupling the MFA model results with the fractions of the substances found in plastic products from Aurisano et al.^49^ and their potential presence in plastic polymers in different application sectors according to Wiesinger et al.,^8^ covering 232 different substances in total. When fractions were given as a range with high and low estimates, an average value was used.

### Data Collection

Trade quantities for plastic products were calculated using the harmonized system (HS) data provided by Statistics Norway (Statistisk sentralbyrå (SSB)),^50^ by calculating the net import in each year and by estimating the amount of each plastic polymer that these quantities contain. The data covered in this study span a period from 2000 to 2020. This temporal focus was chosen since reliable trade data were available and since most plastic products have a mean lifetime that can be covered by this time span, hence giving a good representation of the physical buildup of the present economy. The data consist of trade and production data as well as TCs for each individual year. The input data and the polymer composition for LDPE, HDPE, PP, PS, PVC, EPS, and PVC were adapted from Abbasi et al.^34^ The polymer composition for PUR, PA, PC, and ABS in traded quantities was modeled according to Klotz and Haupt^28^ without any modifications since the Norwegian plastic economy is largely similar to the Swiss one. The mass distributions for these polymers to the different product categories were taken from Liu and Nowack^30^ with slight adjustments to account for the differences in the covered product categories (see Section S3.1 in SI1 for details). Rubber and CA were considered in only tires and cigarettes, respectively. Details on the product categories, the transfer coefficients, and the lifetime distributions are provided in SI1, while the composition of the polymers in the covered HS codes and the list of additives can be found in Supporting Information 2 (SI2). Furthermore, we derived the leaching rates as annual rates that are applied to all materials residing in stocks at the start of each period (year) by dividing the loss rate by the mean product lifetime (Table S9 in SI1).

### Modeling Package

The modeling was carried out using the DPMFA package that was first introduced by Kawecki et al.^33^ The input data, TCs, model compartments, and lifetime distributions were organized in Excel sheets and then fed into the Python-coded model. The package was modified to account for the lifetime-leaching approach. The relevant data and Python code used in this study are made available on Zenodo (10.5281/zenodo.10514261).

## Results

### Norwegian Plastic Cycle

**Figure 1:**
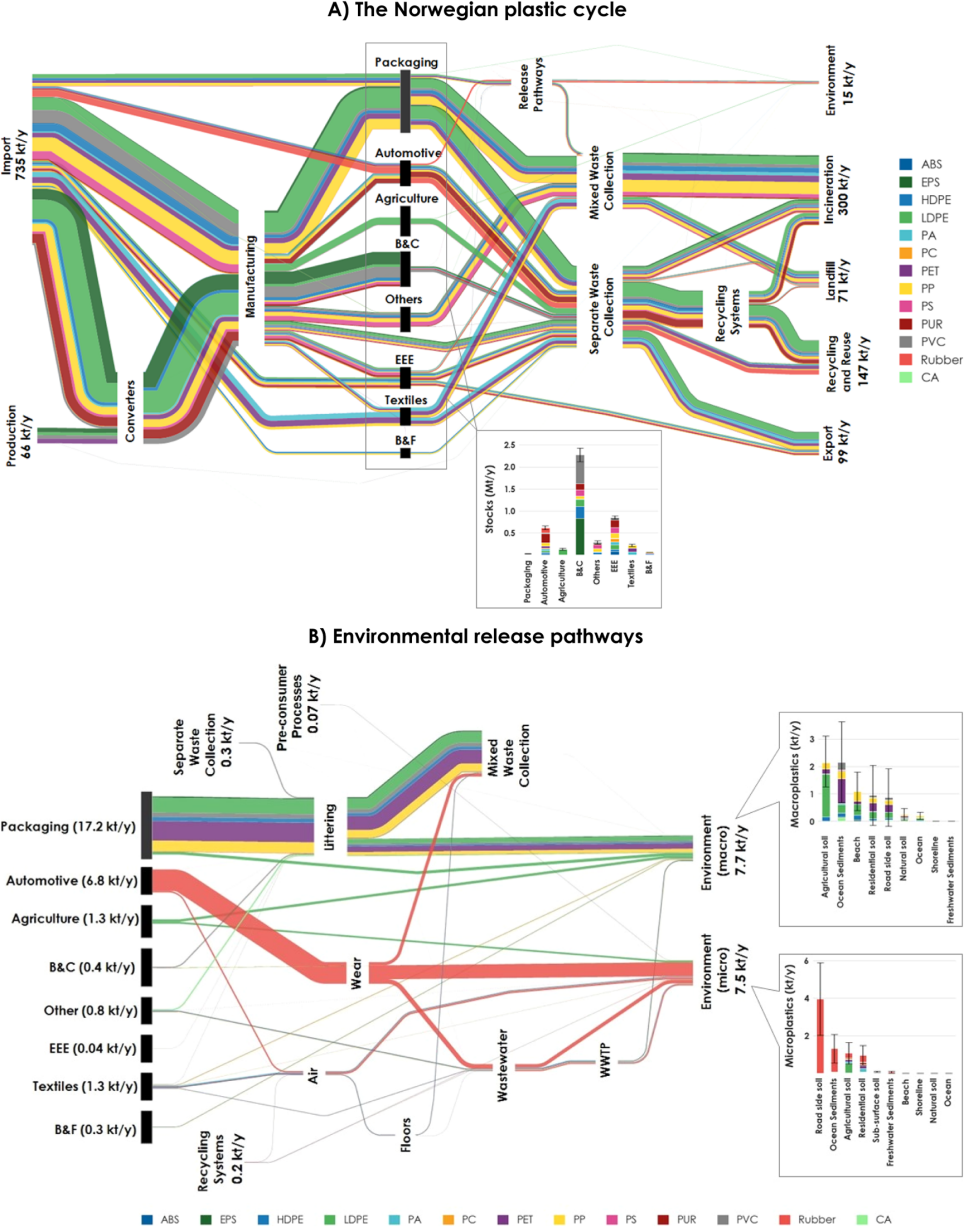
The Norwegian plastic cycle in 2020 for 13 polymer types. A) Sankey diagram of the major flows and stocks. Flows smaller than 1 kt/year are not shown. The length of the black boxes is not representative of the stocks. The quantities in in-use stocks are shown in the box below Figure 1A. Processes were aggregated for better visual presentation. The aggregation of product categories follows the distribution found in Table S1 in SI1, with an exception for clothing, household textiles and technical textiles all being aggregated under “Textiles”. All intermediate release pathways (shown in Table S1) are aggregated in one process. All environmental sinks are aggregated under “Environment”. Separate Waste Collection includes all other waste collection processes other than Mixed Waste Collection. Recycling systems include Packaging recycling, Construction and demolition waste recycling, Agriculture recycling, Large automotive parts, ASR, and WEEP. Recycling and reuse cover Material reuse, Automotive part reuse, and Textile reuse, which act as sinks and are not further considered in this study (i.e., flows cannot be reintroduced to the system from one year to the next). B) Detailed environmental release pathways and distribution between compartments. B&C: Building and Construction; B&F: Boats and Fisheries; EEE: Electrical and Electronic Equipment.

In total, 760 ± 200 kt of plastics was introduced to the Norwegian economy in 2020 (Figure 1), corresponding to 140 ± 36 kg/capita. Packaging accounts for the largest share, with LDPE and PP being the two dominant polymers. Plastics in in-use stocks in 2020 amount to 4470 ± 330 kt (830 ± 60 kg/capita), with the majority residing in the B&C sector. EPS and PVC make up most of plastics residing in use. Approximately 590 ± 110 kt of plastics was released from the stocks in 2020, equivalent to 110 ± 20 kg/capita, of which packaging makes up the largest share with LDPE and PP as dominant polymers. Section S9.2 in SI1 shows a summary of plastics introduced to the market, in-use, and exiting use.

**Figure 2:**
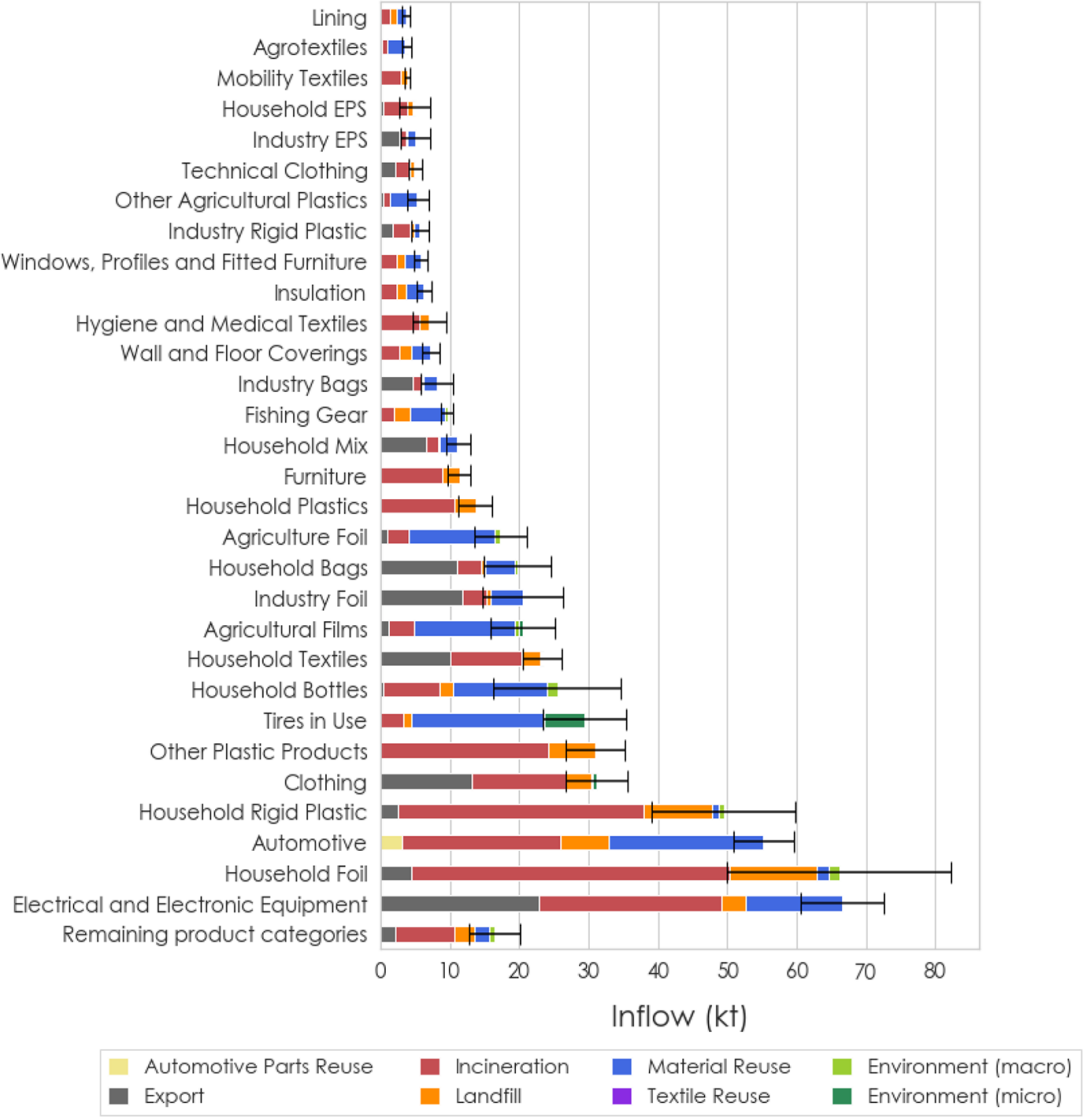
Whereabouts and destinations of the top 30 individual product categories in 2020. The 19 remaining product categories are aggregated under “Remaining product categories”.

**Figure 3:**
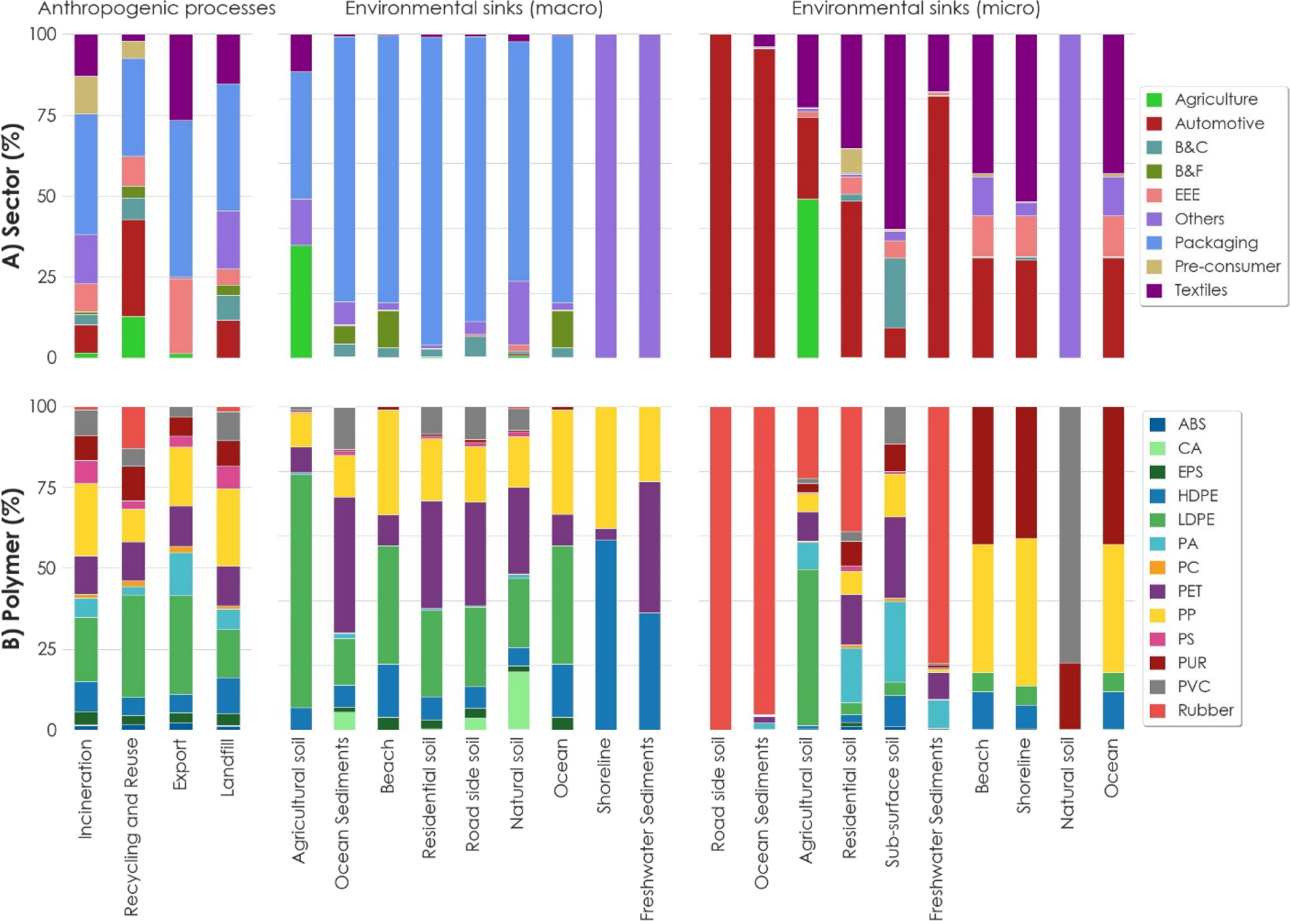
(A) Shares of sectors in the anthropogenic and environmental sinks for macro- and microplastics. (B) Shares of the polymer types for the same processes and sinks. Material reuse, automotive part reuse, and textile reuse are aggregated under Recycling and Reuse. B&C: building and construction; B&F: boats and fisheries; EEE: electrical and electronic equipment.

Around 632 ± 120 kt of plastics entered the anthropogenic and environmental sinks in 2020, of which the majority were incinerated (47.4%) and 15.2 ± 9 kt (2.4%) were released to the environment; see Figure 1A and Figure 2 for a detailed breakdown. Packaging products make up a large share of plastics sent to incineration, while exported plastics consist primarily of textiles, EEE, and packaging products (Figure 3A). Plastics sent to landfills and recycling and reuse originate from diverse application sectors, but packaging products make up a remarkable share of plastics entering these two anthropogenic sinks. Out of the total amounts released to the environment, 7.7 ± 6 kt (1.43 ± 1 kg/capita) was released as macroplastics and 7.5 ± 4 kt (1.39 ± 0.7 kg/capita) as microplastics. Since 2000, 125 ± 21 kt of macroplastics and 117 ± 14 kt of microplastics were released to the environment (Figures S20 and S21 in SI1).

**Figure 4:**
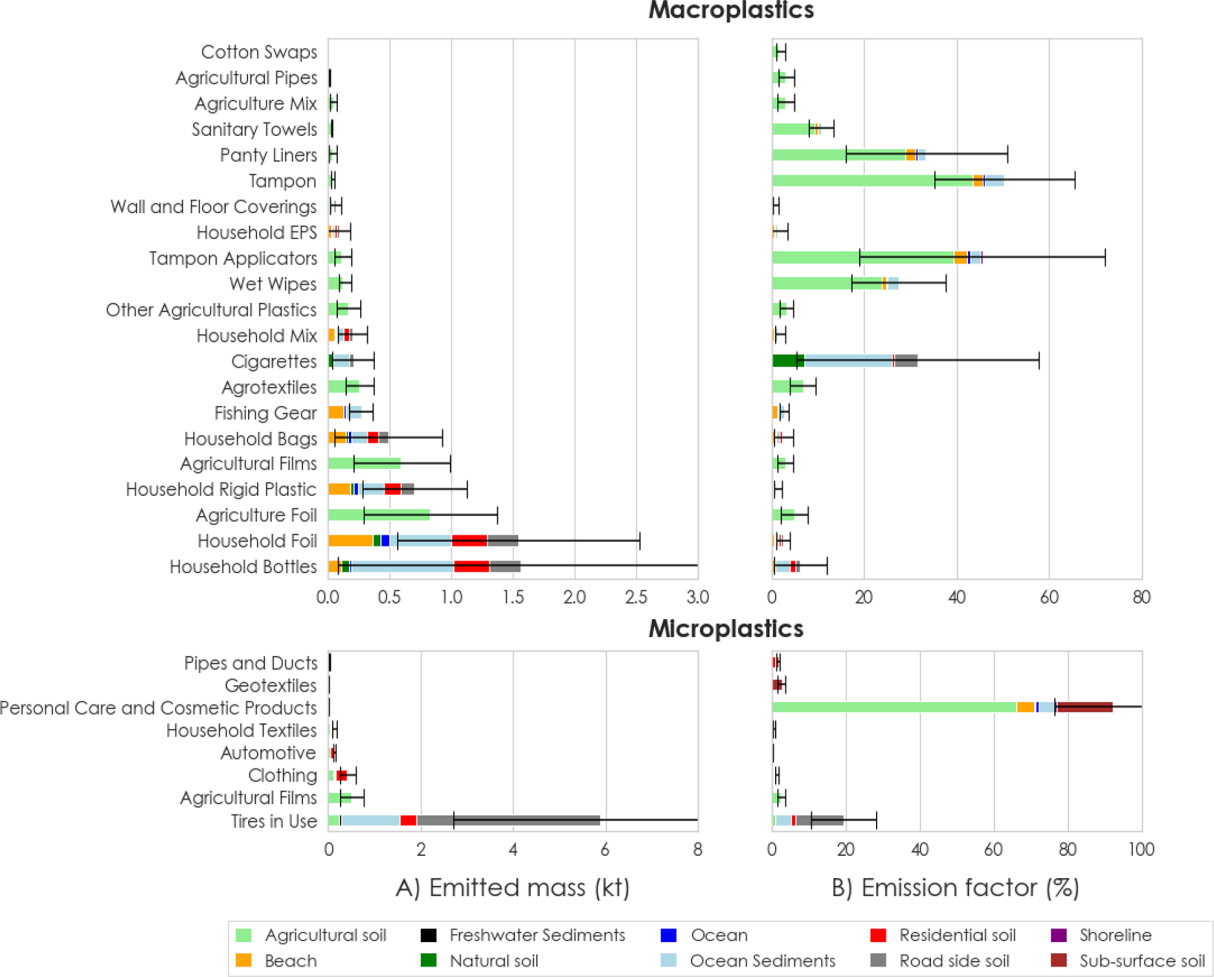
(A) Absolute emitted mass (kt) of selected individual product categories to the environmental sinks and (B) emission factors (%) distinguished for macro- and microplastics in 2020. Emissions factors were calculated as the total inflow into each environmental sink divided by the total outflow leaving the stocks for each product category.

The majority of macroplastics were released to agricultural soils and ocean sediments (Figure 1B). Packaging is responsible for more than two-thirds of the total released macroplastics, with LDPE, PET, and PP being the biggest contributors to these releases and consisting mostly of household bottles, foils, rigid plastics, and bags, of which high fractions entered the marine compartments (Figure 4A). Agricultural foils and films as well as agrotextiles were released in the highest amounts from agricultural applications, mainly to agricultural soils. Flushed products were primarily released into agricultural soils as tampon applicators, panty liners, and wet wipes. CA from cigarettes was also released in high amounts, with a large fraction reaching ocean sediments. For macroplastic releases, tampon applicators, tampons, wet wipes, panty liners, and sanitary towels, as well as cigarettes, show the highest emission factors, while packaging products and fishing gear have the lowest emission rates (Figure 4B).

Almost half of all microplastics was released to roadside soils, and a significant share reached ocean sediments. Microplastics mainly stem from the wear and tear of tires, and rubber is the dominating polymer in these releases (Figure 4a). Clothing, household textiles, and geotextiles were major sources of microplastics released from textile applications, primarily from the use phase. Microplastics leaching from synthetic consumer textiles combined amount to 640 ± 250 t, while all agricultural plastics in total are responsible for 580 ± 275 t of microplastics, with agricultural films being the major contributors. Product categories showing the highest microplastic emission factors were cosmetics and tires, while textile applications show low emission factors compared to all other product categories (Figure 4b).

### Plastic Additives

**Figure 5:**
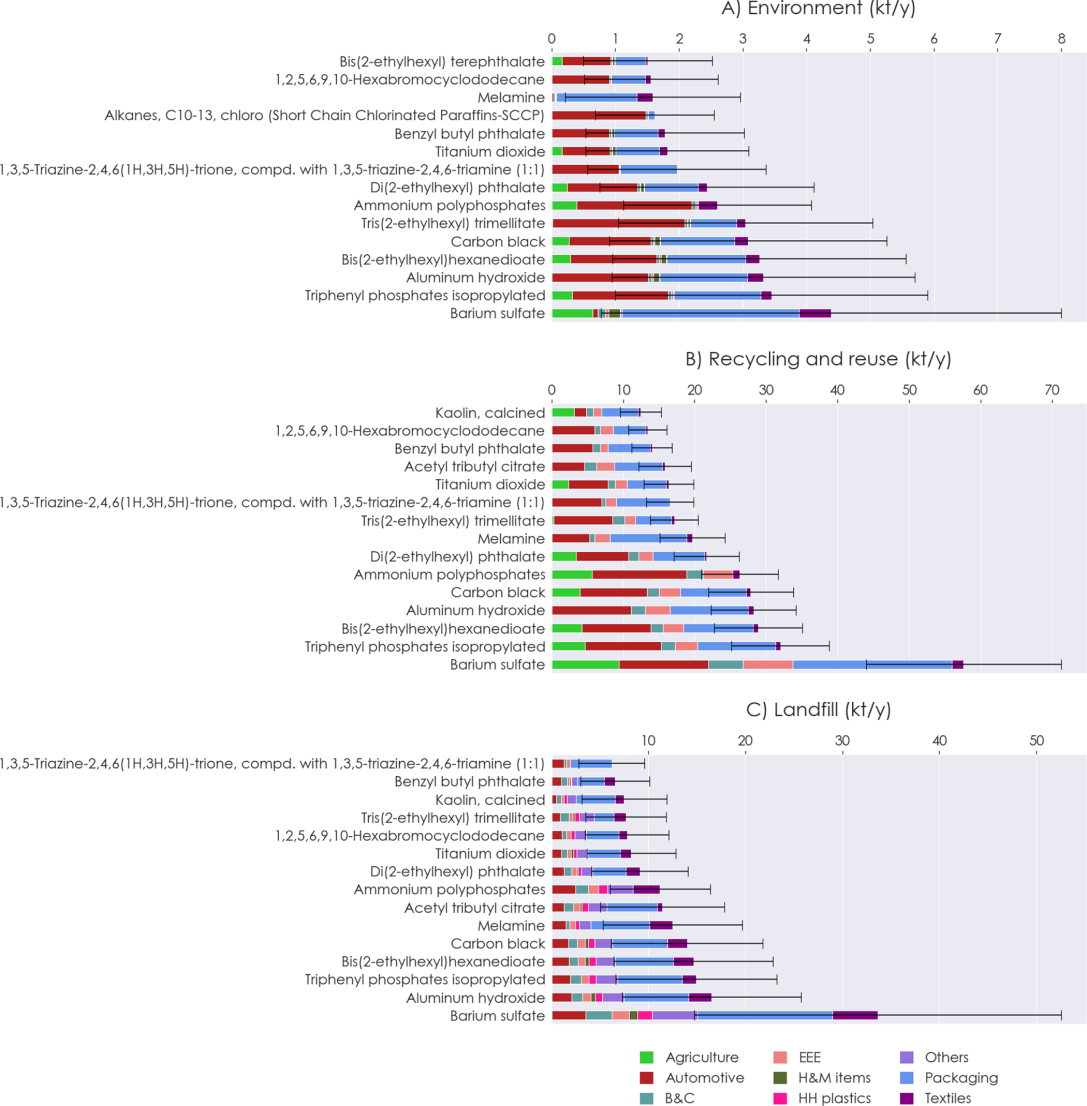
Maximum potential inflow amounts of the top 15 plastic additives per application sector in 2020 to (A) environmental sinks, (B) recycling and reuse processes, and (C) landfill. B&C: building and construction; EEE: electrical and electronic equipment; HH Plastics: household plastics; H&M items: hygiene and medical items.

In 2020, 75 ± 50 kt of additives was potentially contained in plastic products that were released to the environment in Norway, 730 ± 150 kt was included in plastics sent to recycling and reuse, 380 ± 200 kt was included in those that were landfilled, 1430 ± 350 kt was contained in incinerated plastics, and 540 ± 110 kt was included in the exported plastic amounts; see Figure 5 and Figure S24 in SI1 for the responsible application sectors and Figure S25 for the associated polymers, while the detailed results of all 232 substances can be found in SI2. Most of the amounts ending up in the environment as well as those sent to recycling and reuse are caused by the automotive and packaging sectors (Figure S26 in SI1). Rubber, LDPE, and PET make up most polymers that are associated with additives being lost to the environment, while substances found in LDPE, PUR, and PET dominate the recycling and reuse amounts (Figure S27). Packaging, textiles, and other plastic products are responsible for the highest amounts of additives being landfilled, with PVC, PP, LDPE, and PET as main associated polymers.

## Discussion

### Model Limitations and Performance

Our study provides a detailed assessment of the plastic cycle in Norway, and this approach can also be used for other coastal countries. However, several limitations exist, linked to data availability, modeling choices, and simplifications.

Lack of data and varying data quality are the main limitations of our study and contributed to the uncertainty of the model results. This is largely due to the use of varied data sources and the assumptions taken. The absence of comprehensive data meant that we could only include a fraction of the more than 13,000 documented additives.^9^ In addition, information about the exact combination of additives used in different plastic applications is absent. Various substances can be used for the same function (e.g., flame retardant or plasticizer), but our method was unable to account for this aspect (i.e., we assumed that multiple substances can be present in plastics simultaneously even if they serve the same functional purpose). Additionally, we lacked the information regarding the share of products that contain each substance since not all plastic products in each product category have the same mixture of substances. Overall, our assumptions regarding the additives’ estimation have led to an overestimation of the quantities, and the results are therefore only a representation of the maximum potential amounts of the covered substances, rather than the actual amounts.

The chosen temporal scope was also constrained by the unavailability of reliable historical trade data, resulting in an underestimation of the stock and outflow amounts in certain sectors (e.g., B&C), where several product lifetimes can reach up to 80 years. Our system lacks the inclusion of several important microplastic sources, such as aquacultural applications, artificial turfs, and marine coatings and paintings because of a lack of data, and this may have resulted in an underestimation of the overall microplastic releases from the Norwegian economy in our assessment. Another implication of lacking data was the exclusion all possible leaching processes and pathways present in the plastic cycle, such as those related to boats and fishing gear. We applied constant leaching rates in our model, despite the fact that leaching rates have been documented to increase as products age, for example, in garments^51^ and ropes.^52^ The temporal aspect was also neglected in our simplified redistribution model for plastics in the aquatic compartments, i.e., the transport duration for plastic released to a certain environmental compartment to another one is not considered.

The results of this study were benchmarked against previous estimates for validation, including Norwegian studies but also studies with different geographical and temporal scopes (Tables S15–S17 in SI1). We calculated consistently higher plastic amounts present in the Norwegian plastic cycle than Abbasi et al.^34^ for 2020 (see Table S15). This is primarily due to the wider coverage of polymer types in our system. Syversen et al.^53^ and Systemiq^54^ lacked details on product categories and did not differentiate polymer types, hence explaining the differences to our estimates. Deshpande et al.^55^ presented a detailed assessment of plastics used in commercial fishing activities using a quasi-stationary MFA model, and our lower estimates for the loss of fishing gear to the ocean in comparison can be explained by our lack of detailed product categories. Although Systemiq^54^ presented an estimation for the released amounts to the environment (10 kt in 2020), their study lacks specific pathways for the releases and does not differentiate between macro- and microplastic releases. Schwarz et al.^32^ also provided a rough estimate for the released amounts from the Norwegian economy, but their assessment for marine releases does not consider land-based sources and has no specific release pathways for coastal regions. Our results serve as the first detailed assessment of the environmental releases from the Norwegian plastic economy. Additionally, we highlight the importance of distinguishing between the different receiving environmental compartments when mapping the release of plastics for specific world regions.

In comparison to the European average,^33^ the Norwegian population has higher per capita consumption figures (+21%) and plastics found in in-use stocks (+16%). Our estimates also indicate more plastics being released to the environment from the Norwegian anthroposphere in comparison to Switzerland,^29,30^ equivalent to approximately double the amounts of the total per capita flows. These larger quantities can potentially be explained by the lower per capita consumption figures (−21%) present in the Swiss case compared to the Norwegian average.^27^ Sieber et al.^35^ calculated a lower per capita release (−16%) of tire wear rubber from the Swiss economy. This larger Norwegian per capita release could be linked to the lower population density and higher vehicle use per capita.^56,57^ Compared to the Chinese economy,^31^ our results show lower per capita estimates for the releases to the environment.

We estimated higher per capita amounts of di(2-ethylhexyl) phthalate (DEHP) and hexabromocyclododecane (HBCD) being released to the environment from the Norwegian plastic cycle in all cases compared to previous estimates in different geographical scopes (see Table S17 in SI1).^39,40,58,59^ This is mainly due to the differences in the methodologies employed to estimate these amounts and the way in which these releases are defined. The release of additives in previous additive cycle studies is commonly depicted as the amounts that are directly released to the environmental compartments and are often quantified using emission factors specifically linked to the polymer content and corresponding to various life cycle stages.^40,44^ It is important to note that these assessments lack the incorporation of the released additive quantities in association with plastic leakages. While our results highlight the additives’ quantities within emitted plastic polymers, they are only representative of the quantities that are available to be released from plastics rather than those directly emitted.

Our combined lifetime-leaching approach allowed for the combination of two different release mechanisms in a dynamic MFA model. This is crucial since not modeling the leaching of microplastics can lead to an overestimation and underestimation of the current and future releases, respectively, and this approach is necessary in the case of rapid changes in the inflow and stock quantities.

### Norwegian Plastic and Additive Cycles

The large demand for plastic packaging explains the dominance of this sector in the plastic cycle. Packaging products mainly consist of single-use items that have a short lifetime, and this is reflected in their high presence in the waste streams and the low amounts retained in in-use stocks. The majority of plastic packaging is incinerated for energy recovery, which contributes to the release of carbon dioxide and other harmful substances that require a careful treatment.^60^ Only 19% of packaging waste is recycled, mostly with mechanical recycling, which is only feasible for specific polymers such as PET.^61^ Maximum achievable mechanical recycling rates are limited,^62^ and the implementation of other technologies such as chemical recycling is essential for achieving higher recycling rates. The packaging sectors is dominated by olefin-based polymers (e.g., LDPE, HDPE, and PP), which are very difficult to recycle or upcycle even by chemical processes, mainly due to their chemical structure.^63^ Recycling these polymers is also uncompetitive under current market conditions compared to producing virgin polymers.^63^ Additionally, packaging often contains composites with nonplastic materials, which contaminate recycling processes and pose a challenge for the quality of recycled plastics.^64^ However, novel technologies have shown the possibility of upcycling olefin-based polymers to high-value chemicals such as aldehydes and surfactants,^65^ even in the presence of highly contaminated plastic wastes.^66^ Products in the B&C, EEE, and automotive sectors are rather durable with longer lifetimes, hence explaining the large amounts found in in-use stocks. High plastic quantities are likely to arise as waste from these sectors in the future, and closing their material loops is difficult mainly due to their diverse polymer use and product design.^67^

Our results demonstrate that in Norway, plastic flows to the environment are small compared to the amounts that are sent to recycling or waste treatment, reinforcing that proper waste management systems are key in reducing plastic leakage. However, the high consumption of product categories such as packaging products is the main driver for their domination in macroplastic flows, given their low emission factors. Their releases are primarily caused by littering and linked to sweeping efficiencies with high fractions reaching the marine environment. Collected citizen science data through beach-clean ups showed that consumer and food packaging are among the most found items along the Norwegian coasts.^68,69^ A high prevalence of PE followed by PP and PS has been detected in macroplastic samples from Norwegian coastal soils,^70^ indicating agreement with our results for beach macroplastics. Our results indicate the dominance of tire wear rubber in the released microplastic amounts, and similar trends have been documented in previous studies.^17,30,35,71^ This is mainly driven by the combination of high in-use tire stocks and higher leaching rates (2.5–7.5%) compared to other product categories. Tire wear can be amplified by several factors.^72^ High instant torque^73^ and heavier vehicle weight^74,75^ tend to produce more wear. This is more prevalent in electric and hybrid vehicles as opposed to conventional ones.^76^ We expect an underestimation of the released amounts of rubber microplastics since the electric and hybrid passenger vehicle fleets have been surging in Norway. Tire type (i.e., summer or winter tires) also plays a crucial role.^72,76,77^ Capturing released tire wear particles, as opposed to, e.g., released microfibers from clothing, is difficult due to their immediate release to nearby soils. Subsequently, rubber particles are transported to the marine environment through road water runoff and wastewater treatment plant effluents. Once in the marine environment, rubber particles accumulate in marine sediments due to their high polymer density, but due to limitations in available analysis methods, the testing for tire wear rubber has been so far absent in field experiments.^78^ Synthetic textiles have also shown significant amounts of released microplastics, even reaching agricultural soils. The presence of nonagricultural plastics in agricultural soils can be explained by the high rate of sewage sludge application. Agricultural plastic applications are also another source of macro- and microplastic pollution, accumulating in agricultural soils.

All plastic products contain additives, and our results show that large additive quantities are reaching recycling processes. Additives affect the quality of recycled plastics^63^ and may contribute to elevated additive concentrations in the plastic cycle in the long run.^79^ High additive amounts are also entering the environment alongside plastics. Again, and linked to their overall released plastic amounts, tires and packaging are the categories that contributed to the largest quantities of additives in emitted plastics. Additives present in tire rubber have shown exceptionally high toxic levels.^80^ Additionally, several additives released in significant amounts in our assessment have previously demonstrated adverse effects on the biota. Phthalate esters (e.g., DEHP) are endocrine disruptors,^81−83^ while the exposure to organophosphate esters (e.g., triphenyl phosphates isopropylated and triphenyl phosphate) and HBCD have proven to cause reproductive and developmental effects.^84−87^ Additives can leach out from plastics in different lifecycle stages and conditions,^15^ including recycling^88,89^ and landfilling.^90,91^ The continuous release of certain additives such as phthalate esters^92^ and HBCD^87^ can lead to their bioaccumulation in biota. Several additives (e.g., BPA and triphenyl phosphate) have been identified in various Norwegian environmental samples.^93^ Limit-exceeding concentrations of chlorinated paraffins (e.g., short chain chlorinated paraffins, SCCP), linked to the ingestion of plastic particles, have been detected in the livers of herring gulls (*Larus argentatus*) in Northern Norway,^94^ while high concentrations of PBDEs were correlated with the mortality of Northern fulmars (*Fulmarus glacialis*) after consuming plastics.^95^

### Strategies for a More Sustainable Norwegian Plastic Cycle

Understanding the plastic and additive cycles serves as a first step in assessing and improving the sustainability performance of plastic products. The Norwegian Ministry of Climate and Environment presented the nation’s overall strategy to counteract plastic pollution.^96^ Our model allowed us to identify the major polluting sources in the Norwegian plastic cycle, and we therefore emphasize the following strategies that are consistent with our findings to be prioritized for achieving the most effective pollution prevention:

1. Consumption reduction of plastics in certain product categories would yield the largest decrease in macroplastic pollution, especially in highly pollutive sources that exhibit low emission factors, such as consumer packaging and agricultural products. Reduction strategies are often absent in current policies, which mostly focus on increased postconsumer efficiencies (e.g., reduced littering) or address only a narrow set of product types (e.g., tax schemes for carrier bags).^96^ Plastic alternatives have shown higher environmental impacts in comparison to plastics,^97,98^ but these assessments neglected various end-of-life plastic-associated impacts, such as entanglement,^99^ toxic effects from additives,^100^ or microplastic physical impacts.^101^ This is mainly because these impact models are not yet included in environmental assessment frameworks (e.g., life cycle assessment).^102^ However, the relevance of these impact categories^99−101^ highlights that holistic assessments are needed before drawing conclusions regarding the environmental sustainability of plastics compared to their alternatives.
2. Mitigation measures such as changes in tire and vehicle design (e.g., lighter vehicles), use of alternative materials, and changes in driving behavior^72^ and mobility choices could lead to the highest reductions in tire wear rubber emissions. Minimizing the emission at the source is difficult given that a significant share is instantly released to nearby soils. Regulations and strategies to tackle this source are scarce, mainly limited to road cleaning and runoff water capturing.^96^ Additionally, a tax on vehicle weight^103^ is currently implemented and might contribute indirectly to addressing this source of pollution. Assessing the reuse of rubber from tires in artificial turfs is also crucial since this is a common end-of-life treatment option in Norway^96^ and a major source of land-based microplastic emissions.^22^
3. Improving sewage sludge treatment to capture higher amounts of plastic residues prior to agricultural soil application would contribute to emission reductions from several sources such as flushed products, which are primarily released through this pathway. Simultaneously, this could mitigate a considerable fraction of tire wear rubber and textile microfiber emissions.
4. Expanding current policies for mitigating microfiber emissions from synthetic textiles, for instance by increasing the efficiency of washing machine filters.^96^ Our study demonstrates that synthetic textiles have very low emission factors, highlighting the necessity for using alternative materials or changing washing habits to achieve the largest reductions.
5. Regulating and monitoring the application of toxic additives in plastics to reduce their overall impacts is of utmost importance. More knowledge is needed regarding their use patterns, behavior, properties, and their impacts on ecosystems and humans.

## Supporting Information Available

Supporting Information 1: Detailed methods, parameters, model assumptions, and additional figure results (components of the MFA model, leakage pathways, distribution of data quality indicators, annual plastics entering use, in-use stocks, and leaving use per application sector and polymer type, absolute inflow amounts of individual product categories, absolute emitted mass of microplastics and emission factors, cumulative and annual plastic release of macroplastics and microplastics, maximum potential inflow amounts of the top 15 plastic additives, source of total additive amounts, polymer types associated with the total additive amounts, relative uncertainty for the inflows per compartment) (PDF)
Supporting Information 2: Detailed model processes, model data, and additive results (XLSX)
