# Supplementary material for: Mapping Plastic and Plastic Additive Cycles in Coastal Countries: A Norwegian Case Study
Source: Environ Sci Technol. 2024 May 4;58(19):8336–48. doi: 10.1021/acs.est.3c09176 (PMC11097394; doi:10.1021/acs.est.3c09176)
Supplement: Supplementary file 1 — es3c09176_si_001.pdf [file es3c09176_si_001.pdf]

Supporting Information 1 for:

Mapping Plastics and Plastic Additives Cycles in Coastal Countries: A Norwegian Case Study

Ahmed Marhoon[1*], Miguel Las Heras Hernandez[2], Romain Guillaume Billy[1], Daniel Beat Müller[1], Francesca Verones[1]

1 Industrial Ecology Programme, Department of Energy and Process Engineering, Norwegian University of Science and Technology (NTNU), Trondheim, NO-7034, Norway

2 The Climate and Environmental Research Institute (NILU), Trondheim, NO-7013, Norway

* Corresponding author: ahmed.marhoon@ntnu.no

Number of pages in supporting information: 41

Number of figures: 29

Number of tables: 17

Contents

S1 System definition 4

S2 Model processes 4

S3 Transfer Coefficients (TCs) 6

S3.1 Plastics in the anthroposphere 6

S3.2 Plastic release and accumulation 6

S3.2.1 Initial plastic release 6

S3.2.1.1 Macroplastics 7

S3.2.1.1.1 On-the-go consumption of consumer items 7

S3.2.1.1.2 Agriculture 8

S3.2.1.1.3 Automotive 8

S3.2.1.1.4 Dumping 8

S3.2.1.1.5 Flushing rates 9

S3.2.1.1.6 Loss of fishing gear 9

S3.2.1.1.7 Cigarettes littering rate 9

S3.2.1.1.8 Sweeping efficiency 9

S3.2.1.1.9 Macroplastic release to the marine environment and freshwater 9

S3.2.1.2 Microplastics 9

S3.2.1.2.1 Lifetime dependent flows 9

S3.2.1.2.1.1 Microplastics from personal care and cosmetic products 10

S3.2.1.2.2 Leaching dependent flows 10

S3.2.1.2.2.1 Clothes and household textiles 11

S3.2.1.2.2.1.1 Washing 11

S3.2.1.2.2.1.2 Drying and wear 12

S3.2.1.2.2.2 Tires 12

S3.2.1.2.2.3 Other leaching sources 12

S3.2.1.2.3 Microplastic release to the marine environment and freshwater 13

S3.2.1.3 Wastewater management 14

S3.2.1.3.1 Storm water collection 14

S3.2.1.3.2 Wastewater collection 14

S3.2.1.3.3 Sludge 14

S3.2.1.4 Release to outdoor and indoor air 14

S3.2.1.4.1 Outdoor air 14

S3.2.2 Redistribution of initial releases 14

S4 Product lifetimes 15

S5 Combined lifetime-leaching approach 15

S6 Input data 16

S6.1 Packaging, EEE and automotive 16

S6.2 Tires 17

S6.3 Flushed products 17

S6.4 Cigarettes 17

S6.5 Fishing gear 17

S7 Plastic additives quantification 17

S8 Uncertainty analysis 18
S9 Additional results 18
S9.1 Data quality 18
S9.2 Inflows to stocks, in-use stock amounts, and outflows from stocks 19
S9.3 Absolute inflow amounts to sinks and emission factors per individual product category 25
S9.4 Cumulative and annual inflows into the environmental compartments 28
S9.5 Additives 30
S9.6 Comparison to previous studies 34
S9.7 Relative uncertainty 37
S10 References 39

S1 System definition

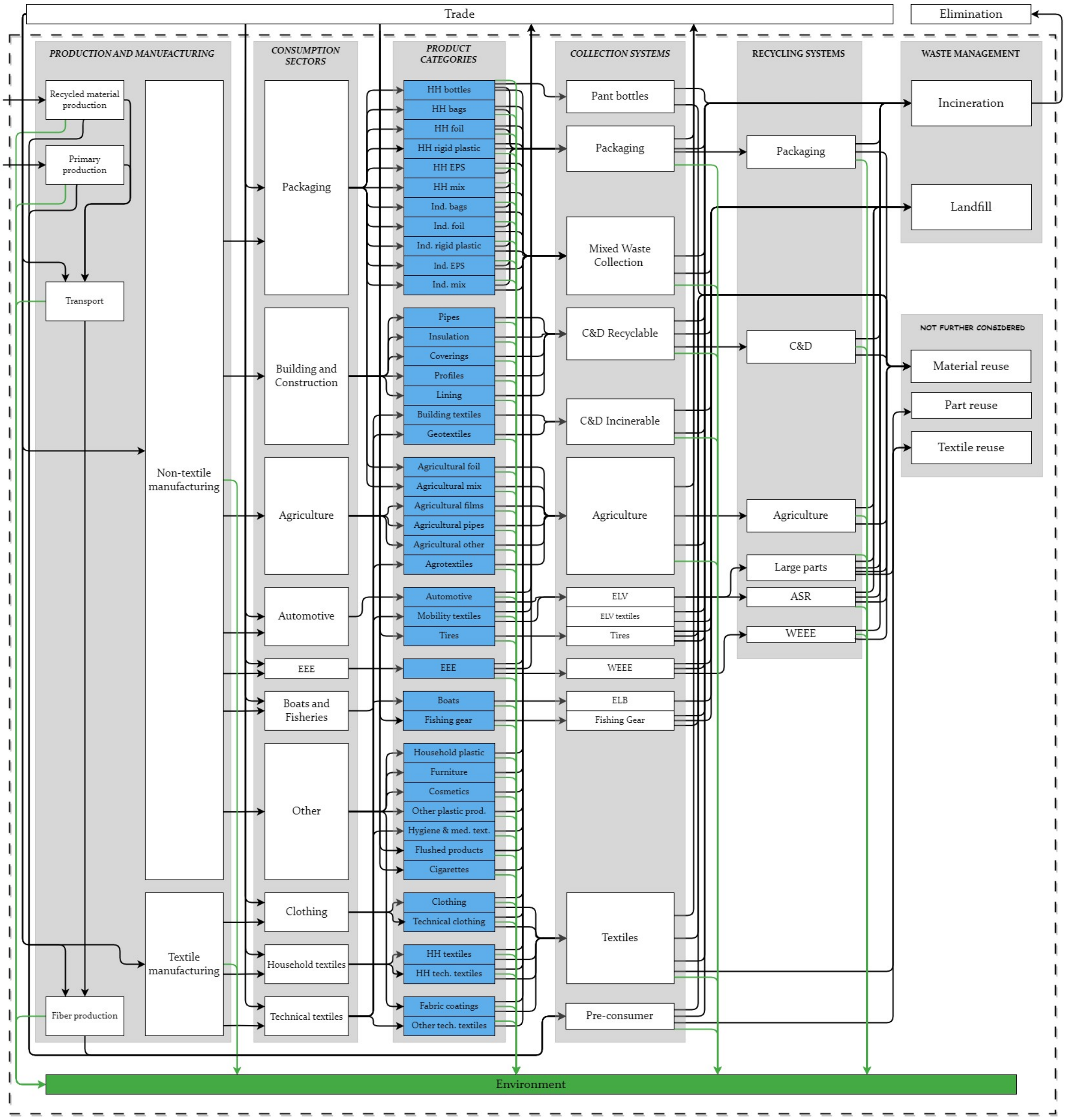

Figure S1. Components of the Material Flow Analysis (MFA) model from Abbasi et al.[1] and modified for this study to include the pathways for plastic leakage to the environment. Stocks are depicted in blue. Green arrows: environmental flows. ASR: auto-shredder residues; C&D: construction and demolition; EEE: electrical and electronic equipment; ELB: end-of-life boats; ELV; end-of-life vehicles; HH: household; Ind.: industrial; WEEE: waste of electrical and electronic equipment.

S2 Model processes

The model proposed by Abbasi et al.[1] was adopted as a base model for plastics in the anthroposphere with slight adjustments. Fishing gear were modeled individually in this study instead of a fraction of technical textiles as assumed by Abbasi et al.[1] Additionally, processes that describe the release of plastics to the environment were added to this model. Eight additional product categories were introduced: Tires, cigarettes, as well as flushed products that contain six categories (tampons, tampon applicators, wet wipes, disposable cleaning cloths, cotton swaps, sanitary towels, and panty liners).

Table S1 shows all model processes covered in this study, while a description of the environmental sinks is given in Table S2. Material reuse, Automotive part reuse, and Textile reuse processes act as anthropogenic sinks and are not further considered in this study (i.e., the flows cannot be reintroduced to the system from one year to the next). This assumption was adopted from the base model.[1]

Table S1. Processes considered in the MFA model. ASR: auto shredder residue; EEE: electrical and electronic equipment; ELB: End-of-life boats; ELV: End-of-life vehicles; HH: Household; PWT: primary water treatment; SWT: secondary water treatment; TWT: tertiary water treatment; WEEE: Waste from electrical and electronic equipment; WEEP: Waste of electrical and electronic plastic. WWTP: Wastewater treatment plant. OSSF: On-site sewage facility. CSO: Combined sewer overflow. Pant bottles refer to deposit return system.

Production and manufacturing	Consumption sectors and individual product categories			Waste collection	Recycling system	Release pathways	Sinks
Recycled material production,<br>*Primary production,*<br>*Inventory,*<br>*Fiber production,*<br>*Non-textile manufacturing,*<br>*Textile manufacturing*	**Packaging:**<br>*HH bottles,*<br>*HH bags,*<br>*HH foil,*<br>*HH rigid plastic,*<br>*HH EPS,*<br>*HH mix,*<br>*Industry bags,*<br>*Industry foil,*<br>*Industry rigid plastic,*<br>*Industry EPS,*<br>*Industry mix,*<br>*Agriculture foil,*<br>*Agriculture mix*<br>- **Construction:**<br>Pipes,<br>Insulation,<br>Coverings,<br>Profiles,<br>Lining<br>- **Boats and fisheries:**<br>*Boats*<br>*Fishing Gear*	**Agriculture:**<br>*Agricultural film,*<br>*Agricultural pipes,*<br>*Agricultural other*<br>- **Automotive:**<br>*Automotive Tires*<br>- **EEE:**<br>*EEE*<br>- **Other plastic:**<br>*Household plastic,*<br>*Furniture,*<br>*Cosmetics,*<br>*Other plastic products,*<br>*Fabric coatings,*<br>*Tampons,*<br>*Tampon applicators,*<br>*Wet wipes,*<br>*Disposable cleaning cloths,*<br>*Cotton swaps,*<br>*Sanitary towels,*<br>*Panty liners,*<br>*Cigarettes*	**Clothing:**<br>*Clothing*<br>**Household textiles:**<br>*Household textiles*<br>**Technical textiles:**<br>*Building textiles,*<br>*Geotextiles,*<br>*Agrotextiles,*<br>*Mobility textiles,*<br>*Hygiene and medical textiles,*<br>*Technical clothing,*<br>*Technical household textiles,*<br>*Other technical textiles*	*Pant bottles,*<br>*Mixed waste,*<br>*Packaging waste,*<br>*Construction and demolition recyclables,*<br>*Construction and demolition incinerables,*<br>*Agriculture waste,*<br>*ELV,*<br>*ELV textiles,*<br>*ELB,*<br>*Fishing gear waste,*<br>*WEEE,*<br>*Textile waste,*<br>*Tire waste,*<br>*Pre-consumer waste*	*Packaging recycling,*<br>*Construction and demolition recycling,*<br>*Agriculture recycling,*<br>*Large automotive parts,*<br>*ASR,*<br>*WEEP*	*On the go consumption,*<br>*Dumping,*<br>*Litter in residential environments,*<br>*Litter in roadsides,*<br>*Litter in natural environments,*<br>*Outdoor air,*<br>*Indoor air,*<br>*Indoor floors,*<br>*Industrial stormwater,*<br>*Stormwater,*<br>*Wastewater,*<br>*WWTP,*<br>*OSSF,*<br>*Sludge,*<br>*CSO,*<br>*PWT,*<br>*SWT,*<br>*TWT,*<br>*Wear,*<br>*Tumble drying,*<br>*Indoor cloth-line drying,*<br>*Outdoor cloth-line drying,*<br>*Highways,*<br>*Roads,*<br>*Other roads,*<br>*RRTP,*<br>*Particles collection*	**Anthropogenic:**<br>*Incineration,*<br>*Landfill,*<br>*Material reuse,*<br>*Part reuse,*<br>*Textile reuse,*<br>*Export*<br>**Environmental:**<br>*Agricultural soil,*<br>*Natural soil,*<br>*Residential soil,*<br>*Roadside soil,*<br>*Sub-surface soil,*<br>*Ocean,*<br>*Ocean sediments,*<br>*Beach,*<br>*Freshwater sediments,*<br>*Shoreline*

Table S2. Detailed description of the environmental sinks considered in the MFA model.

Environmental sinks	Description
Agricultural soil	Soil used for agriculture
Natural soil	Soil in rural and non-populated areas
Residential soil	Soil in cities and populated area
Roadside soil	Soil in roadside
Sub-surface soil	Soils below the top later of soils
Ocean	Oceans and seas
Ocean sediments	Floor areas of oceans and seas
Beach	Beach and coastal areas of oceans and seas
Freshwater sediments	Floor areas of rivers, lakes, canals and streams
Shoreline	Shore areas of rivers, lakes, canals and streams

S3 Transfer Coefficients (TCs)

S3.1 Plastics in the anthroposphere

TCs from Abbasi et al.[1] were adopted with no further changes for LDPE, HDPE, PP, PS, PVC, EPS, and PVC. The mass distribution for PUR, PA, PC, and ABS to the different product categories were taken from Liu and Nowack,[2] with slight adjustments for the product categories naming to account for the differences, see Table S3.

Table S3. Assigned product categories due to the different naming between Liu and Nowack[2] and this study.

Product category in this study	Product category in Liu and Nowack[2]
Household Foil	Consumer Films
Household Bottles	Consumer Bottles
Household Bags	Consumer Bags
Household Mix	Other Consumer
Agriculture Foil	Agricultural Packaging Films
Industry Bags	Non-consumer Bags
Industry Foil	Other Non-consumer Films
Industry Mix	Other Non-consumer Packaging

For tires, according to Statistics Norway (SSB),[3] 86% of tires collected from households were sent to recycling, 10% to incineration, and the remaining to landfill in 2020.

S3.2 Plastic release and accumulation

S3.2.1 Initial plastic release

Plastic leakage was modeled following the methodologies presented by Kawecki and Nowack[4] and Sieber et al.[5]. The reader is referred to these studies for detailed modeling approaches. Only the adjustments to the methods to account for the release of plastics for coastal countries are discussed in the sections below.

S3.2.1.1 Macroplastics

The initial release of macroplastics is presented in this section. Figure S2 shows an overview of the release pathways.

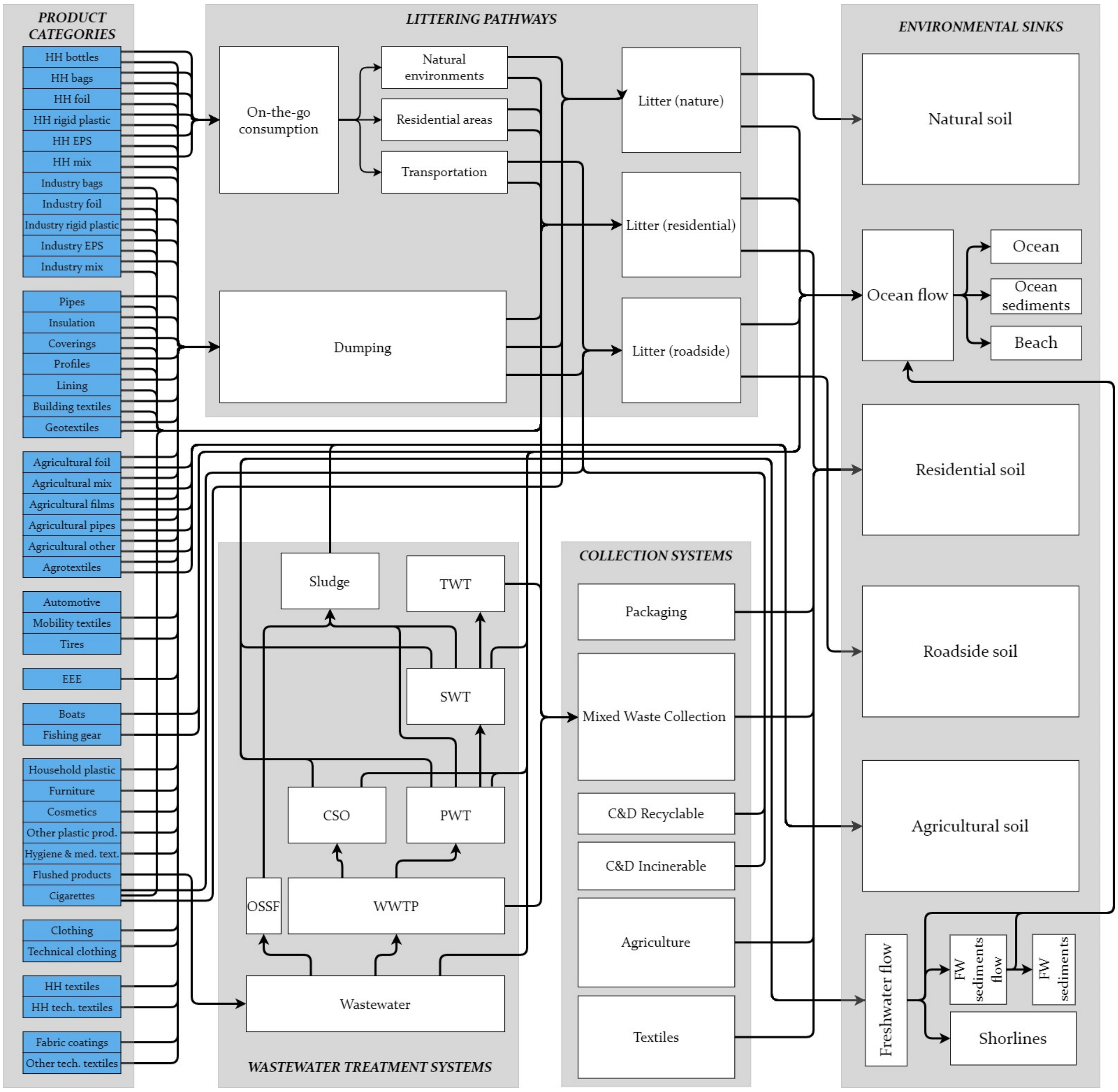

Figure S2. Leakage pathways for macroplastics with all relevant processes employed in the Material Flow Analysis (MFA) model. CSO: combined sewer overflow; C&D: construction and demolition; EEE: electrical and electronic equipment; HH: household; FW: Freshwater; Med.: medical; OSSF: on-site sewage facilities; PWT: primary water treatment; SWT: secondary water treatment; Text.: textiles; TWT: tertiary water treatment; WWTP: wastewater treatment plant.

S3.2.1.1.1 On-the-go consumption of consumer items

The fractions of consumer items used on the go were adjusted to be aligned with the product categories in this model. Table S4 shows the adjusted values and the assumptions taken with regards to the respective polymers with respect to the methodology presented by Kawecki and Nowack.[4]

Table S4. Adjusted fraction of items consumed on the go per polymer with regards to the methodology presented by Kawecki and Nowack[4].

	Plastic polymers										
Product category	LDPE	HDPE	PP	PS	EPS	PVC	PET	PUR	ABS	PA	PC
HH bags	0.1395	0.2411	0*	0.0000	0	0.1903**	0*	0	0	0.1903**	0
HH bottles	0.0000	0.3544***	0.3544***	0	0	0.0000	0.3544	0	0	0	0
HH foil	0.1017	0*	0.1897	0	0	0.2000	0*	0	0	0.1638**	0
HH rigids	0	0.0797**	0.0813	0.0755	0	0*	0.0851	0	0	0	0
HH EPS	0	0	0	0	0.099125**	0	0	0	0	0	0
HH mix	0	0.1176	0.0909	0.0769	0	0.099125**	0.1111	0.099125**	0	0.099125**	0

* Removed (included in Kawecki and Nowack[4] but this polymer share does not exist in this study).

** Average using all polymers values.

*** Assumed to be the same as PET.

S3.2.1.1.2 Agriculture

The fraction for the loss of "Agricultural Mix" product category in this model is assumed to be the same as "Other Agricultural Plastics" in Kawecki and Nowack[4] with a value of 3.8%.

S3.2.1.1.3 Automotive

We assume that no macroplastics are lost from the "Automotive" product category. Losses are only accounted for "tires" and "mobility textiles".

S3.2.1.1.4 Dumping

Kawecki and Nowack[4] assumed that a person is 100 times more likely to discard their waste in public bins rather than dump it in Switzerland. For the Norwegian context, 125 tons of waste were collected in public bins in the city center in Trondheim in 2007[6] and the household waste can be found using Statistics Norway (SSB) data.[3] The population living in the city center is 4783 and in the whole city is 207580 in 2022 (2.3% of the population lives in the city center).[7] We assume that half of the waste found in public bins in the city center originate from people living in the city center (i.e., 62.5 tons). This gives us 2717.4 tons of waste found in public bins for the whole city of Trondheim. We used historical GDP to back-cast the household waste to 2007.[8]

Table S5. Household waste generation in 2007 and 2018 in Trondheim.

Year	Waste (tons)	GDP in 2007[8]
2007	50277.65*	56177.48
2018	62874.7	70252.74

*Back-casted using GDP.

We therefore derive a rate of 0.054% of dumping in Norway, which is double rate for Switzerland derived by Kawecki and Nowack[4]. Dumping is applied to all product categories except "Automotive". We assume that the sinking of boats in the ocean has the same rate as dumping.

S3.2.1.1.5 Flushing rates

Kawecki and Nowack[4] derived flushing probabilities using various studies.[9,10] Moreover, a Norwegian report[11] presented an estimate for flushing rates in 2019. Table S6 summarizes the flushing probabilities used in this study.

Table S6. Derived high and low flushing probabilities per flushing product.

Item	Flushing probability (low)	Flushing probability (high)
Tampon	15%[11]	91.4%[4]
Wet wipes	3%[11]	46%[4]
Panty liner	16.5%[4]	52.5%[4]
Tampon applicator	4.2%[4]	76.4%[4]
Cotton swaps	3.2%[4]	5%[4]
Disposable cleaning cloth	0.9%[4]	
Sanitary towels	6.5%[4]	15%[11]

S3.2.1.1.6 Loss of fishing gear

According to Deshpande et al.,[12] the loss rate of fishing gear to the ocean range between 0.4 – 4.4%.

S3.2.1.1.7 Cigarettes littering rate

We assume a littering rate ranging between 0 and 100%.

S3.2.1.1.8 Sweeping efficiency

We applied a sweeping rate of 90%[11] to the waste littered in residential and road side areas. Littered waste in natural environments is assumed to not to be swept.

S3.2.1.1.9 Macroplastic release to the marine environment and freshwater

Here, we propose a new pathway for the release of littered macroplastics. The fraction of littered macroplastics that reach the marine environment is assumed to be proportional to the coastal population. Jambeck et al.[13] used the populations that live within 50 km from the coastlines to estimate the amount of plastics that is available to reach the marine environment. In Norway, ~92% of the population live within 50km of the coastline.[7] Meijer et al.,[14] estimated that 80% of littered plastics in coastal cities reach the ocean. Combining these two fractions, we therefore derive a rate of 74% of available littered plastics to be released to the ocean in Norway, while the rest is distributed to terrestrial compartments. We further assumed all macroplastics released to freshwater eventually end up in the ocean.

S3.2.1.2 Microplastics

S3.2.1.2.1 Lifetime dependent flows

Figure S3 summarizes the release pathways for lifetime related microplastic releases.

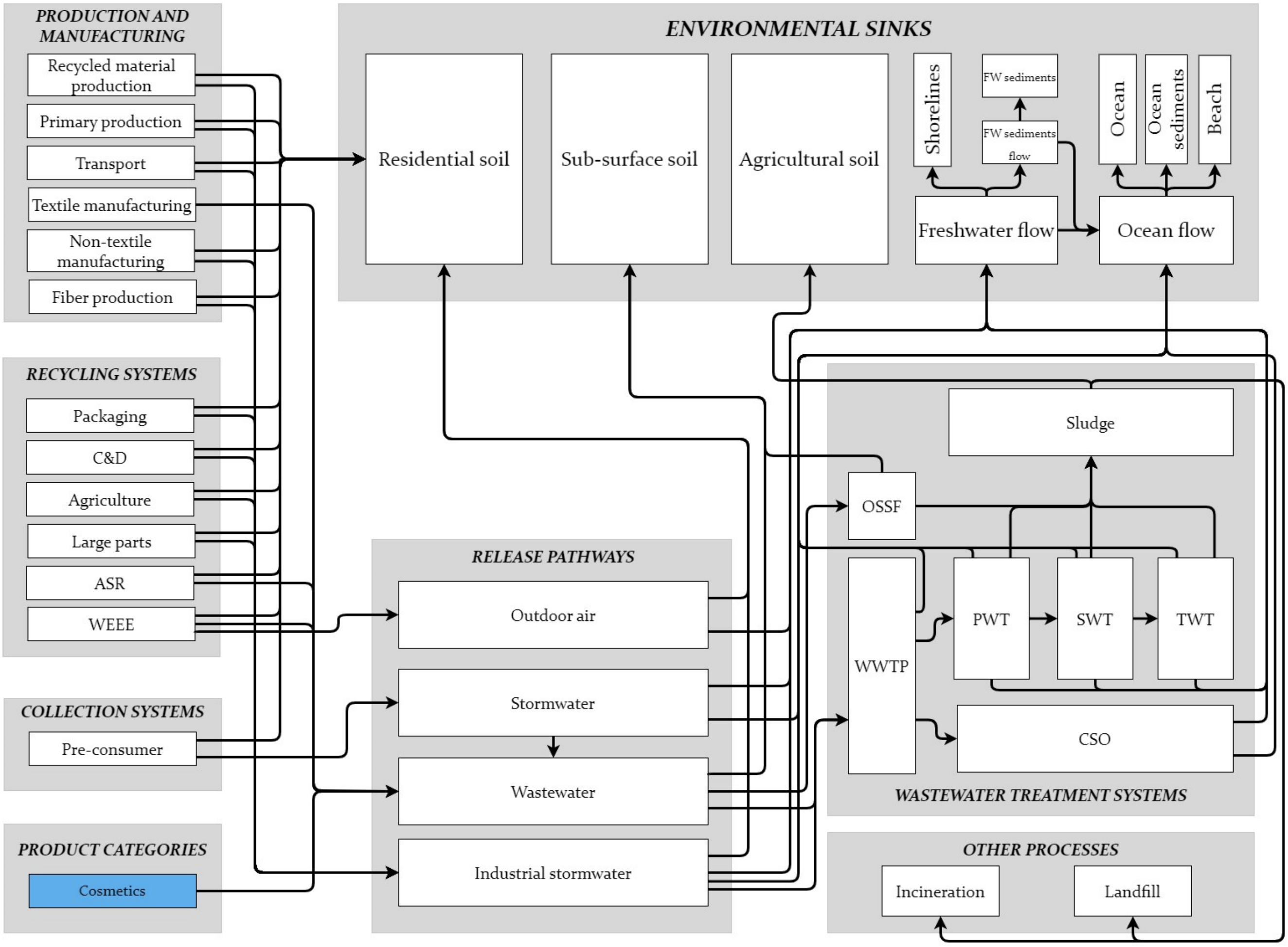

Figure S3. Leakage pathways for lifetime dependent microplastic outflows with all relevant processes employed in the Material Flow Analysis (MFA) model. ASR: auto-shredder residues; CSO: combined sewer overflow; C&D: construction and demolition; OSSF: on-site sewage facilities; PWT: primary water treatment; SWT: secondary water treatment; Text.: textiles; TWT: tertiary water treatment; WEEE: waste of electrical and electronic equipment; WWTP: wastewater treatment plant.

S3.2.1.2.1.1 Microplastics from personal care and cosmetic products

We assume 100% of cosmetic products is released to wastewater.

S3.2.1.2.2 Leaching dependent flows

Figure S4 shows the pathways for the release of leaching related microplastic flows.

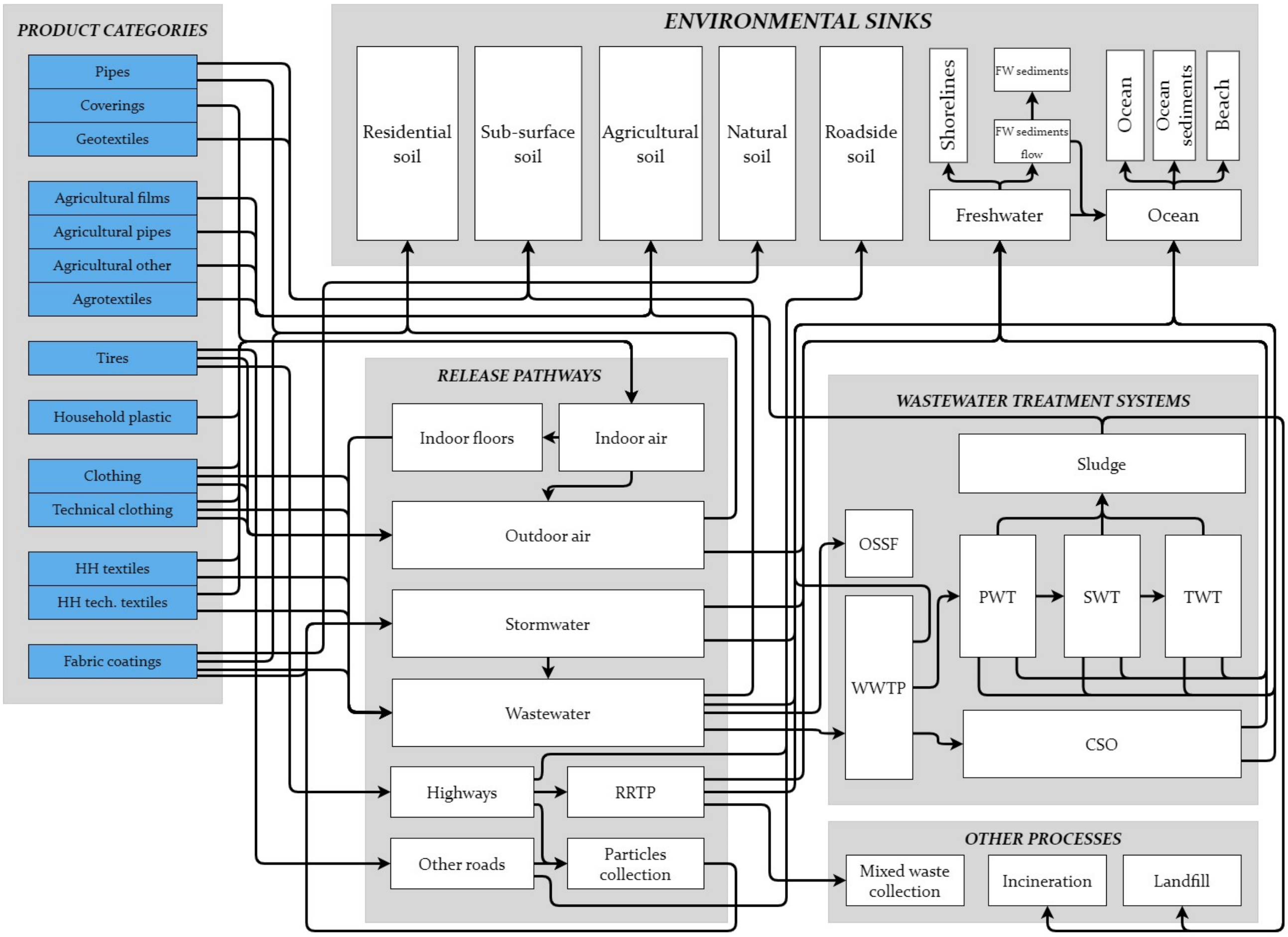

Figure S4. Leakage pathways for microplastic leaching outflows with all relevant processes employed in the Material Flow Analysis (MFA) model.

S3.2.1.2.2.1 Clothes and household textiles

A shedding rate is applied on the product categories "Clothing" and "Technical Clothing", "Household Textiles", and "Household Technical Textiles". The following sections describe the derived leaching rates for washing, drying and wear:

S3.2.1.2.2.1.1 Washing

A leaching rate for shedding of microplastics during wash can be obtained by firstly deriving an annual number of washing cycles. This can be done by using the following relationship:

$$N_{washing} = \frac{Number\ of\ washing\ cycles\ performed\ in\ the\ whole\ lifetime}{mean\ lifetime}$$

According to various studies,[4,15–17] a textile clothing piece is washed on average between 19 and 39 times across its whole lifetime, and household textiles for 5.9 times. The annual leaching rate can then be calculated by multiplying the shedding rate of one washing cycle by the annual number of washing cycles:

$$Annual\ Leaching\ rate = Shedding_{washing,cycle} \times N_{washing}$$

The Plastic Leak Project Report[18] presented high (134 mg/kg), average (46 mg/kg) and low (24 mg/kg) shedding rates based on an extensive literature review. Only high and low shedding rates are chosen in this study.

Table S7. Number of lifetime washing cycles, mean lifetime, and the derived number of washing cycles used in this study.

Product categories	Number of lifetime washing cycles (cycles)	Mean lifetime (years)	Derived annual number of washing cycles (cycles/year)
Clothing	19-39	1.2	15.83-32.5
Technical clothing			
Household textiles	5.9	3.014	3.22-6.61
Technical household textiles			

S3.2.1.2.2.1.2 Drying and wear

The annual number of washing cycles discussed previously was used in the calculation of drying and wear related microplastic emissions as opposed to the total number of washing cycles used by Kawecki and Nowack[4] to derive annual leaching rates.

S3.2.1.2.2.2 Tires

During use, tires release particles due to the forces between the tire and the road surface. An average passenger car losses between 10 – 30% of its tread rubber during its whole lifetime.[19,20] Given that tires have a mean lifetime of 4 years,[21] the annual leaching rate per year for tires is therefore 2.5 – 7.5%. The modeling of tire release follows the methodology presented by Sieber et al.[5] without any further modifications.

S3.2.1.2.2.3 Other leaching sources

The emission rates for the product categories were derived as annual rates by dividing the loss rates presented by Kawecki and Nowack[4] by the mean lifetimes used in this study without any adjustments (see Table S8), while a summary of all emission sources is presented in Table S9.

Table S8. Summary of the leaching rates modified using the loss rates presented by Kawecki and Nowack.[4]

Sector	Product category	Loss rates adopted from Kawecki and Nowack[4] (%)	Mean lifetime (years)	Derived annual leaching rates (%/year)
Building and Construction	Wall and floor coverings	0.1	40	0.0025
	Pipes and ducts	0.1	20	0.005
	Geotextiles	0-1.3	12	0-0.108
Agriculture	Agricultural films	2	1.5	1.33
	Agricultural pipes	0.1	40	0.0025
	Other agricultural plastics	0.1	1.5	0.067
	Agrotextiles	0-1.3	1.5	0-0.867
Other	Fabric coatings	1	5	0.2
Technical textiles	Household plastics	0.1	5	0.02

Table S9. A summary of annual leaching rates derived and used in this study.

Sector	Product category	Process	Receiving process	Derived annual leaching rates (%/year)
Building and Construction	Wall and floor coverings		Indoor air	0.0025
	Pipes and ducts	Wear	Residential soil, sub-surface soil	0.005
	Geotextiles	Wear	Sub-surface soil	0 – 0.108
Agriculture	Agricultural films	Wear	Agricultural soil	1.33
	Agricultural pipes	Wear	Agricultural soil	0.0025
	Other agricultural plastics	Wear	Agricultural soil	0.067
	Agrotextiles	Wear	Agricultural soil	0 – 0.867
Other	Fabric coatings	Wear	Residential soil, natural soil, wastewater, stormwater	0.2
	Household plastics	Wear	Indoor air	0.02
Clothing	Clothing	Washing	Wastewater	0.4355 – 0.037992
	Technical clothing			0.4355 – 0.037992
Household textiles	Household textiles			0.088574 – 0.007728
	Technical household textiles			0.088574 – 0.007728
Clothing	Clothing	Tumble drying	Indoor air, wastewater, mixed waste collection	0.0180066 – 0.0369688
	Technical clothing			
Household textiles	Household textiles		Indoor air, mixed waste collection	
	Technical household textiles			
Clothing	Clothing	Indoor cloth-line drying	Indoor air	0 – 0.074035
	Technical clothing			
Household textiles	Household textiles			
	Technical household textiles			
Clothing	Clothing	Outdoor cloth-line drying	Wastewater	0 – 0.074035
	Technical clothing			
Clothing	Clothing	Wear	Indoor air, outdoor air	0.04 – 0.86
	Technical clothing			
Household textiles	Household textiles		Indoor air	0.0946 – 0.143
	Technical household textiles			
Automotive	Tires	Wear	Outdoor air, highways, other roads	2.5 – 7.5

S3.2.1.2.3 Microplastic release to the marine environment and freshwater

Since 92% of the population live within 50km from the coastline,[7] we assume 92% of this fraction is released into the ocean, while the rest is released to freshwater.

S3.2.1.3 Wastewater management

S3.2.1.3.1 Storm water collection

According to a report by Statistics Norway,[22] there are 39,000 km of wastewater pipes in Norway, of which 7,000 km are joint pipes (wastewater and stormwater) and 32,000 km of only wastewater. Using this information, we assume that the fraction of joint pipes represents the fraction of stormwater that goes to a wastewater treatment plants (17.95%), while the rest is discharged immediately to waterbodies. We therefore assume 92% of this fraction is released into the ocean, while the rest is released to freshwater, since 92% of the population live within 50km from the coastline.[7]

S3.2.1.3.2 Wastewater collection

In Norway, 87% of the population are connected to a wastewater collection system.[22] We assume the remaining 13% go through on-site sewage facilities. 64% of all wastewater undergo advanced treatment (primary, secondary and tertiary stages), 21% only mechanical (primary) treatment, and 2% is discharged without treatment.[22] 2% of the collected wastewater is released to waterbodies without any treatment.[22] The remaining 98% undergo treatment stages according to the method presented by Kawecki and Nowack[4]. Combined sewer overflow amounts to 1-3% in Norway.[22]

S3.2.1.3.3 Sludge

Data on sludge handling in Norway is available.[22] The detailed TCs can be found in Supporting Information 2 (SI2).

S3.2.1.4 Release to outdoor and indoor air

S3.2.1.4.1 Outdoor air

An estimation was done based on land cover for Norway. According to Statistics Norway,[23] 6.2% of Norway consists of inland waters. We assigned this fraction for the amounts in outdoor air to be deposited to freshwater, while the rest to residential soil.

S3.2.2 Redistribution of initial releases

The redistribution of plastics after the initial release for aquatic environments was included in this model. A similar approach was applied by Schwarz et al.[24] This redistribution depends on the density of the polymer, where polymers with densities higher than water are all assumed to sink. High density polymers include PET, PVC, PS, PA, ABS, PC, CA and rubber. Low density polymers include PP, HDPE, LDPE, PUR, EPS. PET is an exception since many plastic bottles and containers are made of PET and can be airfilled if the lid is attached, increasing the floating potential[25]. Schwarz et al.[24] assumed only 89% of PET macroplastics sink, while the rest are either transported, beached, or remain afloat. Kaandorp et al.[26] estimated that 8-12% of plastics in the Mediterranean Sea remained afloat, 49-63% was beached, and 37-51% have sunk. We assume the same fractions are followed for plastics in the ocean and freshwater, except that no plastics remain afloat in freshwater. Furthermore, Hurley et al.[27] found that 70% of stored microplastics in riverbeds get flushed out to the marine environment during flooding events. We assume 70% of all plastics (micro and macro) stored in freshwater sediments are to be flushed into marine environments regardless of the polymer type. The release of plastics from terrestrial compartments to the aquatic environments is possible, especially through flooding events.[28] However, due to lack of evidence this pathway has been neglected in this model.

Table S10. Transfer coefficients for the redistribution in the aquatic compartments.

From	To	Low density polymers	High density polymers	PET macroplastics
Freshwater	Freshwater sediments	37% – 51%	100%	89%
Freshwater	Shoreline	49% – 63%	0	5.4% – 6.9%
Freshwater	Ocean	Rest	0	rest
Freshwater sediments	Ocean	70%	70%	70%
Freshwater sediments	Freshwater sediments	30%	30%	30%
Ocean	Ocean sediments	37% – 51%	100%	89%
Ocean	Beach	49% – 63%	0	5.4% – 6.9%
Ocean	Ocean	8% – 12%	0	0.88% – 1.3%

S4 Product lifetimes

The lifetimes of individual product categories were taken from Abbasi et al.[1] with no adjustments. For tires, a mean lifetime of 4 years is assumed.[21] Flushed products are assumed to have a lifetime similar to packaging products, while cigarettes are assumed to be similar to "other plastics" product category.

S5 Combined lifetime-leaching approach

A basic, inflow-driven dynamic model relies mainly on lifetime functions. However, not all environmental releases of plastics follow this same pattern. For example, some plastic products, such as tires and textiles, exhibit a leaching behavior during their residing time in the stocks (e.g., due to wear and tear during use). This leaching can be accounted for by applying a leaching rate to the amounts residing in the stocks.

Inflow-driven dynamic models rely mainly on lifetime functions that describe the residing time of materials in stocks, which as a result establish scheduled outflows for each individual cohort distributed over a future time period. The outflow and stocks are usually described as follows (Eq. 1):

$$O_{(t,c)} = I_c \times \int_c^t pdf_{(u,c)} du \qquad (1)$$

$$S_{(t,c)} = I_c \times sf_{(t,c)} \qquad (2)$$

Where t is the time, c is the cohort, I_c is the inflow for cohort c, $O_{(t,c)}$ is the lifetime dependent outflows for cohort c in time t, $S_{(t,c)}$ is the mass stored in stocks for cohort c in time t, $pdf_{(u,c)}$ is the probability density function (for the cohort c of exiting the stock at time u), and $sf_{(t,c)}$ is the survival function.

A leaching model is calculating outflows from the stock, usually using a leaching rate constant over time and cohorts (x_f in Eq. 3):

$$O_{(t)} = x_f \times S_{(t)} \qquad (3)$$

These equations were discretized to consider time steps of one year. To account for the amounts lost due to leaching, a correction factor was applied to the lifetime dependent outflows and stocks:

$$O_{LT_{(t,c)}} = (1 - x_f)^{t-c+1} \times I_c \times \int_{c}^{t} pdf_{(u,c)} \mathrm{du} \quad (4)$$

$$S_{(t,c)} = (1 - x_f)^{t-c} \times I_c \times sf_{(t,c)} \quad (5)$$

$$O_{LE_{(t,c)}} = x_f \times (1 - x_f)^{t-c} \times I_c \times sf_{(t,c)} \quad (6)$$

Or

$$O_{LE_{(t,c)}} = x_f \times S_{(t,c)} \quad (7)$$

Where x_f is the annual leaching rate, $O_{LT_{(t,c)}}$ is the lifetime dependent outflows for cohort c during year t, $O_{LE_{(t,c)}}$ is the leaching outflows for cohort c during year t.

S6 Input data

Plastic input data were estimated using the methodology presented by Kawecki et al.[29] Trade quantities for plastic products were calculated using the harmonized system (HS) data provided by Statistics Norway (Statistisk sentralbyrå (SSB))[30] by taking the net import for 2000 to 2020. The plastic content of LDPE, HDPE, PP, PS, PVC, EPS, and PVC were taken from Abbasi et al.,[1] while the shares of PUR, PA, PC, and ABS were taken from Klotz and Haupt.[31] A matrix showing the polymer content in these traded quantities for all studied polymers can be found in SI2.

The polymer composition for packaging, automotive, electrical and electronic equipment (EEE), tires, flushed products, and cigarettes were calculated separately.

S6.1 Packaging, EEE and automotive

We used data from PlasticsEurope for 2020[32] to estimate the polymer composition in traded quantities in packaging, electrical and electronic equipment (EEE) and automotive plastics, shown in Table S11. Additionally, the plastic share of packaging mass that is traded alongside goods was adjusted to 0.76% according to Kawecki et al.[29] instead of the previously used lower end share value of 0.38% in Abbasi et al.[1]

Table S11. Full breakdown of plastic demand per polymer in all sectors in Europe.[32]

	Packaging (%)	Automotive (%)	EEE (%)
LDPE	29.8	3.9	9.2
HDPE	18.0	7.6	5.6
PP	23.4	21.7	16.2
PS	2.9	1.4	5.6
EPS	1.4	0.2	0
PVC	2.0	3.5	4.3
PET	20.6	0	0.3
ABS	0.1	6	7.6

PA	0.3	6	7.3
PC	0.1	4.4	6.3
PUR	0.3	14.1	10.6

S6.2 Tires

Tires can be imported as individual parts or alongside vehicles. For passenger cars, tires make up 3% of the total vehicle mass, and 5% of trucks.[33] The net import of tires as parts and the tires in vehicles and trucks were calculated using the relevant HS codes that can be found in SI2. On average, a tire consists of 40 – 60% rubber (natural and synthetic).[20,34] We neglect the difference between natural and synthetic rubber in this study.

S6.3 Flushed products

Products at high risk of flushing include personal care and hygiene products. Kawecki and Nowack[4] presented a list of items using evidence from previous studies, and they estimated their polymer composition and consumption figures using a bottom-up approach in Switzerland. The derived input data for this model, which were adapted for this study. can be found in the SI2.

S6.4 Cigarettes

Cigarette filters contain cellulose acetate (CA). We assume that filters make up 35% of the cigarette total mass and contain 90% of cellulose acetate while the remaining 10% are additives. The HS codes for cigarettes can be found in SI2.

S6.5 Fishing gear

We assumed the distribution of polymers in the traded quantities according to Deshpande et al.,[12] see Table S12.

Table S12. Distribution of plastic polymers in traded fishing gear.

HS code	Description	HDPE	PP	PA
5607	Twine, cordage, ropes	0.4	0.4	0
5608	Made-up knotted fishing nets of man-made textile materials (excl. landing nets)	0.25	0.25	0.25
	Knotted netting of twine, cordage, rope or cable, by the piece or metre; made-up nets, of man-made textile materials (excl. made-up fishing nets, hairnets, nets	0.25	0.25	0.25

S7 Plastic additives quantification

The mass of additive is calculated as follows (Eq 7):

$$Mass_{additive} = Mass_{polymer} \times Concentration_{additive} \qquad [kt] \qquad (3)$$

Where $Mass_{additive}$ is the total mass of additive, $Mass_{polymer}$ is the total mass of plastic polymer, and $\mathrm{Concentration_{additive}}$ is the concentration of additive in the polymer matrix.

S8 Uncertainty analysis

Table S13. Pedigree matrix with five data quality indicators to account for the uncertainty adopted from Kawecki et al.[35]

Data quality (DQIS)	Very good	Good	Poor	Very poor
Quality indicator	1	2	3	4
Geographical representativeness	Same region (Norway)	Socioeconomically similar region (i.e., Europe)	Socioeconomically different region (e.g., USA)	Socioeconomically very different region (i.e., World)
Temporal representativeness	Same year	Time difference of 1-5 years	Time difference of 6-10 years	Time difference of more than 10 years
Material representativeness	Same polymer	Same polymer datum corrected with data for all polymers	Data for a different polymer or for plastic as a whole or including similar materials	Including non-similar materials
Completeness	Includes all relevant processes/flows	Includes main processes/flows	Partially including main processes/flows	Important processes/flows are missing
Source reliability	Official report or peer reviewed documentation	Market reports and other reports. Public database	Qualified estimate	Non-qualified estimate

S9 Additional results

S9.1 Data quality

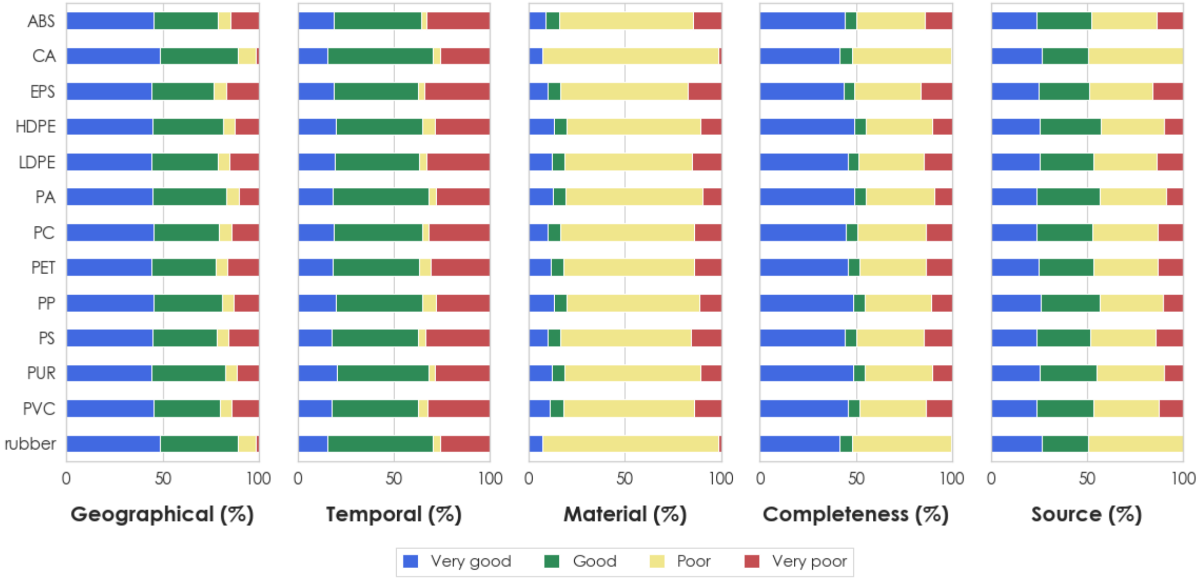

Figure S5. Distribution in (%) of data quality indicators used in this study for all processes per polymer type in 2020.

S9.2 Inflows to stocks, in-use stock amounts, and outflows from stocks

Table S14. Comparison of plastics entering use, in-use stocks, and leaving use per application sector and polymer type in 2020.

	Entering use			In-use stocks			Leaving use		
Sector	*kt*	*%*	*kg/capita*	*kt*	*%*	*kg/capita*	*kt*	*%*	*kg/capita*
Packaging	238.4 ± 52	31.4	44.4 ± 10	23.8 ± 5	0.5	4.4 ± 1	237.1 ± 63	40.2	44.2 ± 12
Agriculture	32.8 ± 16	4.3	6.1 ± 3	133.7 ± 23	3	24.9 ± 4	26.2 ± 7	4.4	4.9 ± 1
B&C	148.4 ± 46	19.6	27.6 ± 9	2275.9 ± 157	50.9	424.0 ± 29	24.9 ± 3	4.2	4.6 ± 0.5
Automotive	88.6 ± 18	11.7	16.5 ± 3	619.2 ± 39	13.9	115.4 ± 7	84.6 ± 8	14.3	15.8 ± 1
EEE	90.2 ± 16	11.9	16.8 ± 3	845.7 ± 38	18.9	157.5 ± 7	66.6 ± 11	11.3	12.4 ± 2
Textiles	81.3 ± 25	10.7	15.2 ± 5	223.2 ± 29	5	41.6 ± 5	80.7 ± 14	13.7	15.0 ± 3
B&F	12.3 ± 1	1.6	2.3 ± 0.2	60.3 ± 3	1.4	11.2 ± 1	9.7 ± 0.4	1.6	1.8 ± 0.1
Others	66.1 ± 23	8.7	12.3 ± 4	285.4 ± 39	6.4	53.2 ± 7	59.5 ± 8	10.1	11.1 ± 2
Polymer	*kt*	*%*	*kg/capita*	*kt*	*%*	*kg/capita*	*kt*	*%*	*kg/capita*
ABS	11.7 ± 2	1.5	2.2 ± 0.3	106.8 ± 4	2.4	19.9 ± 1	10.1 ± 1	1.7	1.9 ± 0.2
CA	0.7 ± 0.1	0.1	0.1 ± 0	2.7 ± 0.2	0.1	0.5 ± 0	0.6 ± 0.4	0.1	0.1 ± 0.1
EPS	66.2 ± 11	8.7	12.3 ± 2	845.8 ± 26	18.9	157.6 ± 5	17.8 ± 5	3	3.3 ± 1
HDPE	62.2 ± 17	8.2	11.6 ± 3	426.5 ± 34	9.5	79.5 ± 6	47.0 ± 9	8	8.8 ± 2
LDPE	157.3 ± 46	20.8	29.3 ± 9	437.3 ± 51	9.8	81.5 ± 10	139.0 ± 33	23.6	25.9 ± 6
PA	41.5 ± 7	5.5	7.7 ± 1	178.7 ± 10	4	33.3 ± 2	38.5 ± 4	6.5	7.2 ± 1
PC	10.8 ± 2	1.4	2.0 ± 0.3	100.1 ± 4	2.2	18.6 ± 1	8.8 ± 1	1.5	1.6 ± 0.2
PET	78.2 ± 18	10.3	14.6 ± 3	160.4 ± 19	3.6	29.9 ± 4	72.6 ± 16	12.3	13.5 ± 3
PP	123.0 ± 34	16.2	22.9 ± 6	422.3 ± 40	9.5	78.7 ± 8	111.2 ± 23	18.9	20.7 ± 4
PS	46.8 ± 12	6.2	8.7 ± 2	360.9 ± 28	8.1	67.2 ± 5	30.8 ± 4	5.2	5.7 ± 1
PUR	53.8 ± 14	7.1	10.0 ± 3	527.9 ± 34	11.8	98.4 ± 6	47.3 ± 6	8	8.8 ± 1
PVC	77.3 ± 29	10.2	14.4 ± 5	785.0 ± 71	17.6	146.2 ± 13	36.4 ± 8	6.2	6.8 ± 1
rubber	28.5 ± 6	3.8	5.3 ± 1	112.9 ± 10	2.5	21.0 ± 2	29.4 ± 5	5	5.5 ± 1

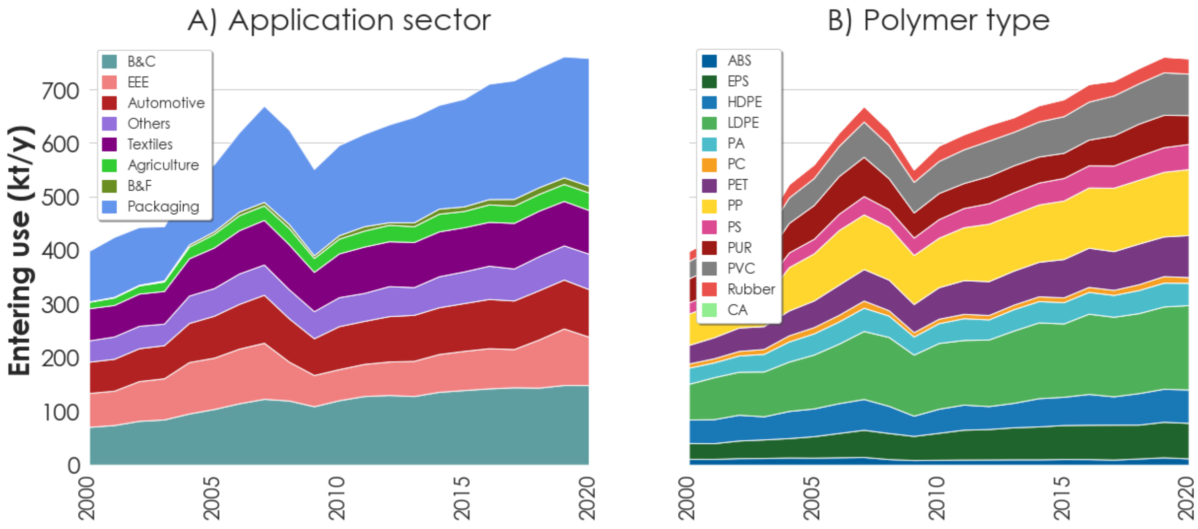

Figure S6. Annual plastics entering use per A) application sector, and B) polymer type between 2000 and 2020.

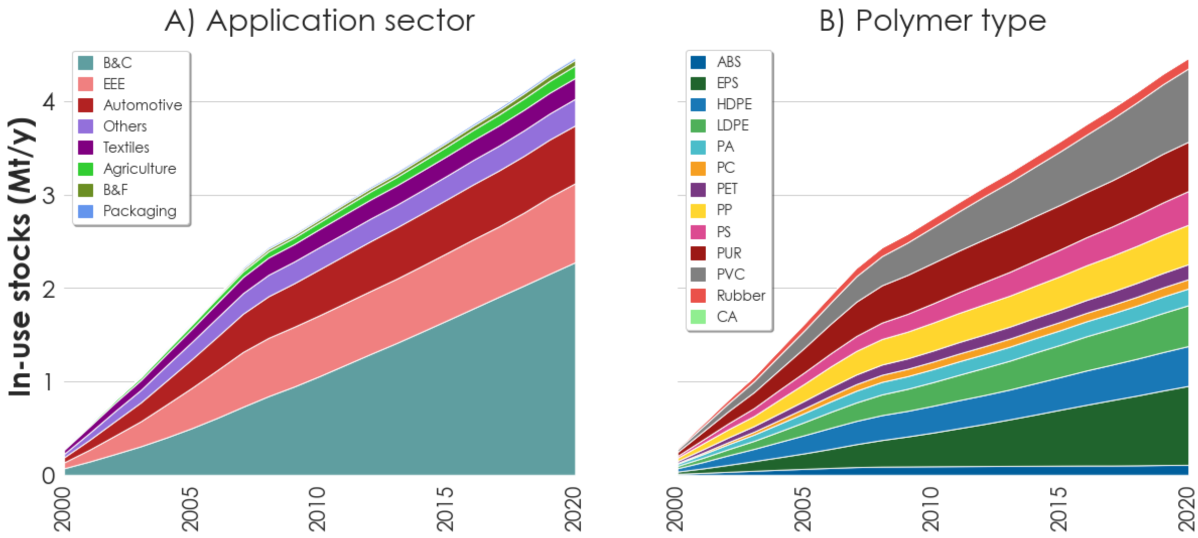

Figure S7. Annual plastics in-use stocks per A) application sector, and B) polymer type between 2000 and 2020.

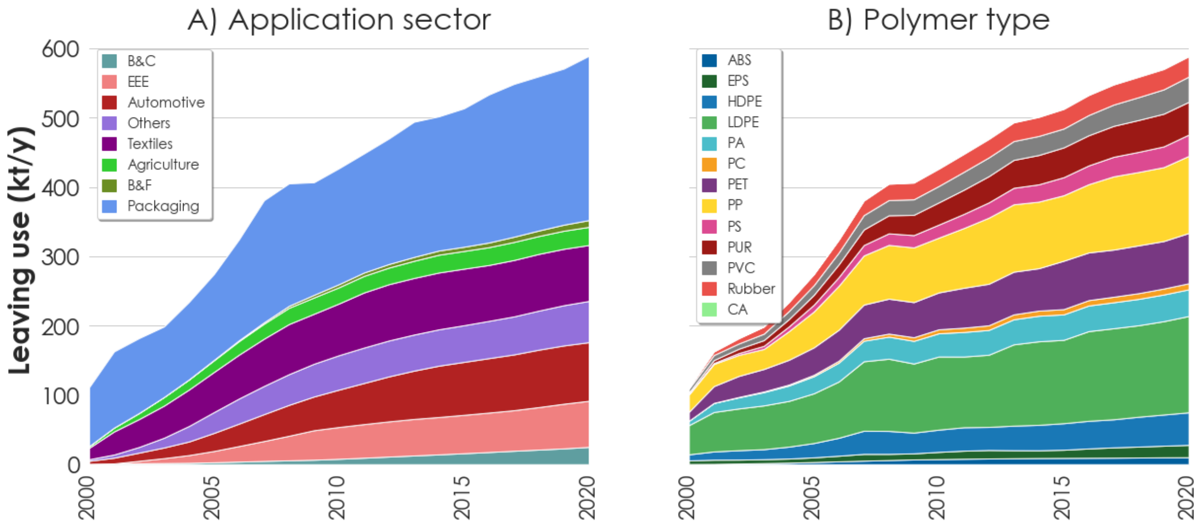

Figure S8. Annual plastics leaving use per A) application sector, and B) polymer type between 2000 and 2020.

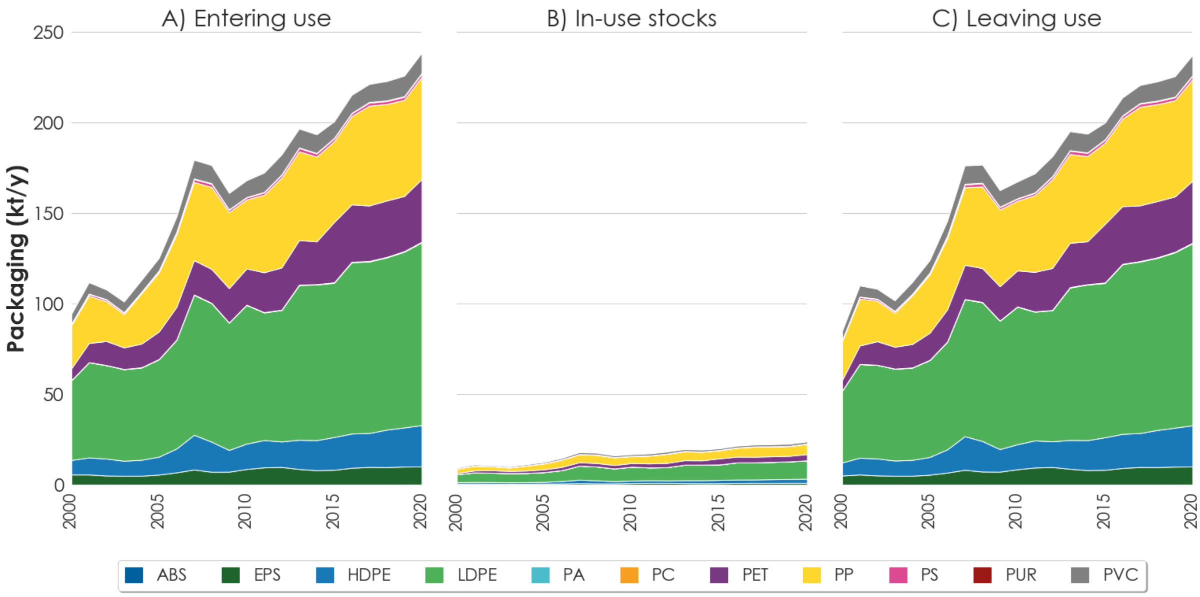

Figure S9. Annual plastics A) entering use, B) in-use stocks, and C) leaving use, distinguished for the polymer type between 2000 and 2020 for the packaging sector.

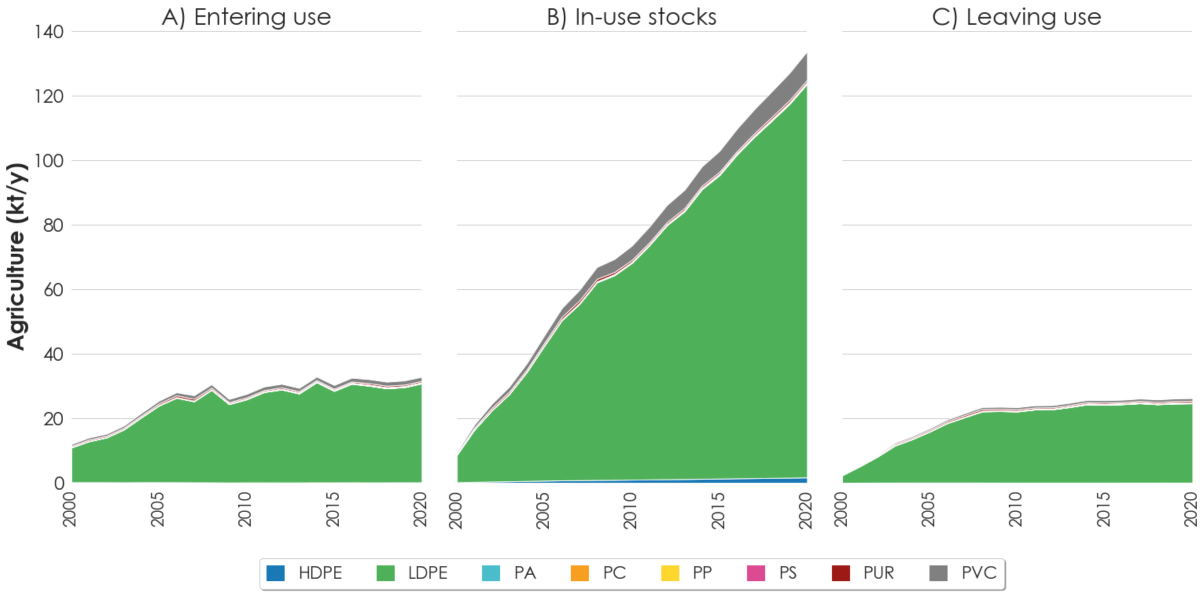

Figure S10. Annual plastics A) entering use, B) in-use stocks, and C) leaving use, distinguished for the polymer type between 2000 and 2020 for the agricultural sector.

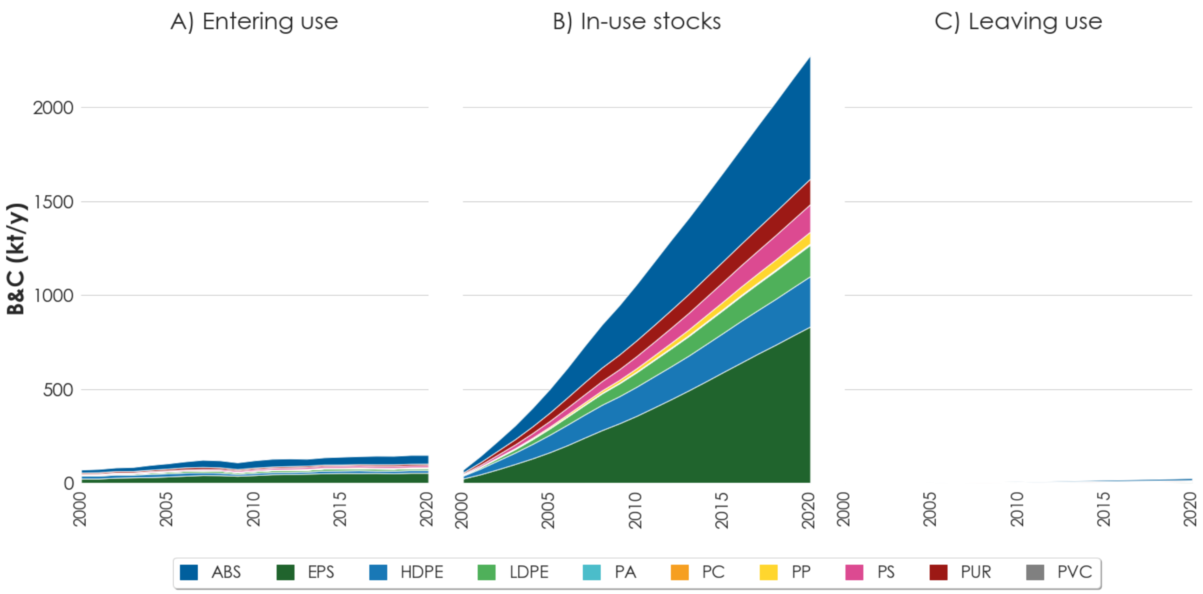

Figure S11. Annual plastics A) entering use, B) in-use stocks, and C) leaving use, distinguished for the polymer type between 2000 and 2020 for the building and construction (B&C) sector.

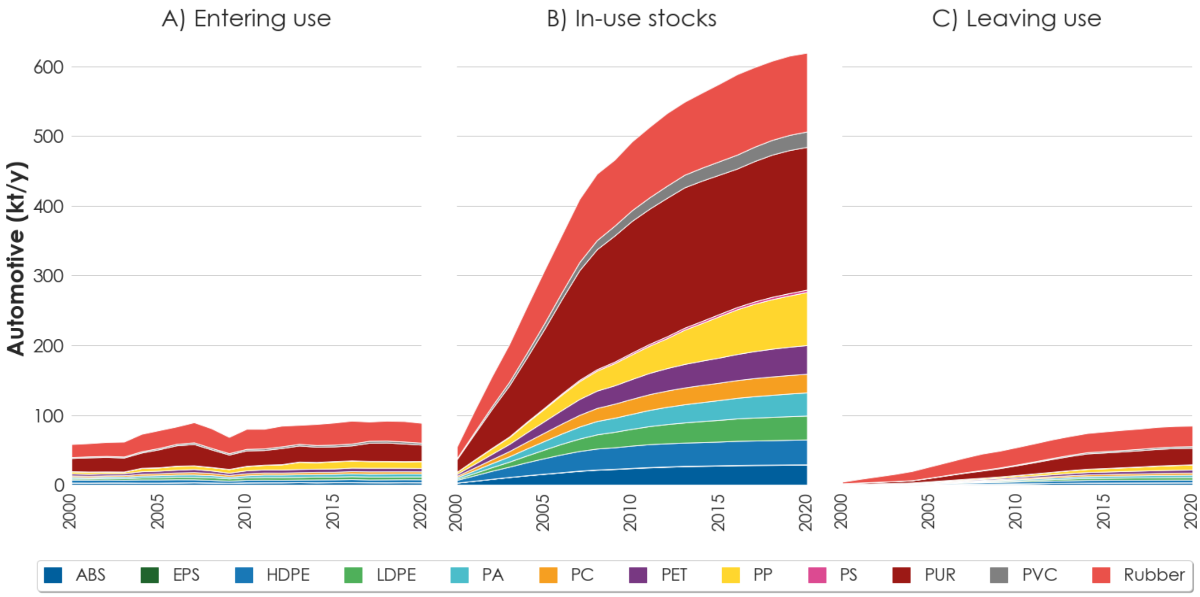

Figure S12. Annual plastics A) entering use, B) in-use stocks, and C) leaving use, distinguished for the polymer type between 2000 and 2020 for the automotive sector.

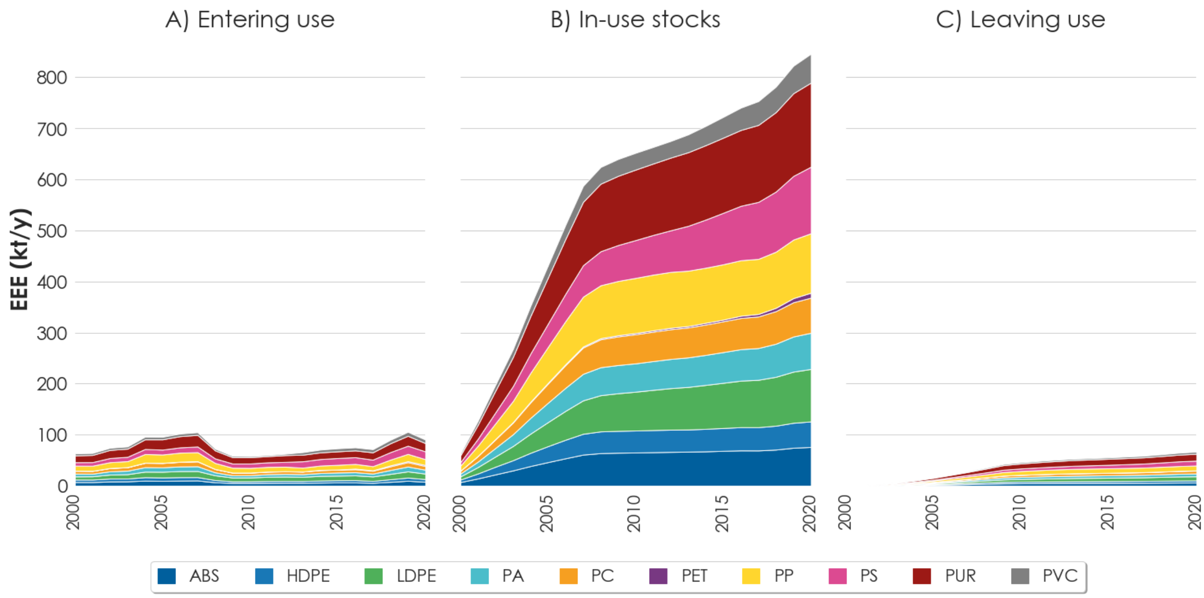

Figure S13. Annual plastics A) entering use, B) in-use stocks, and C) leaving use, distinguished for the polymer type between 2000 and 2020 for the electrical and electronic equipment (EEE) sector.

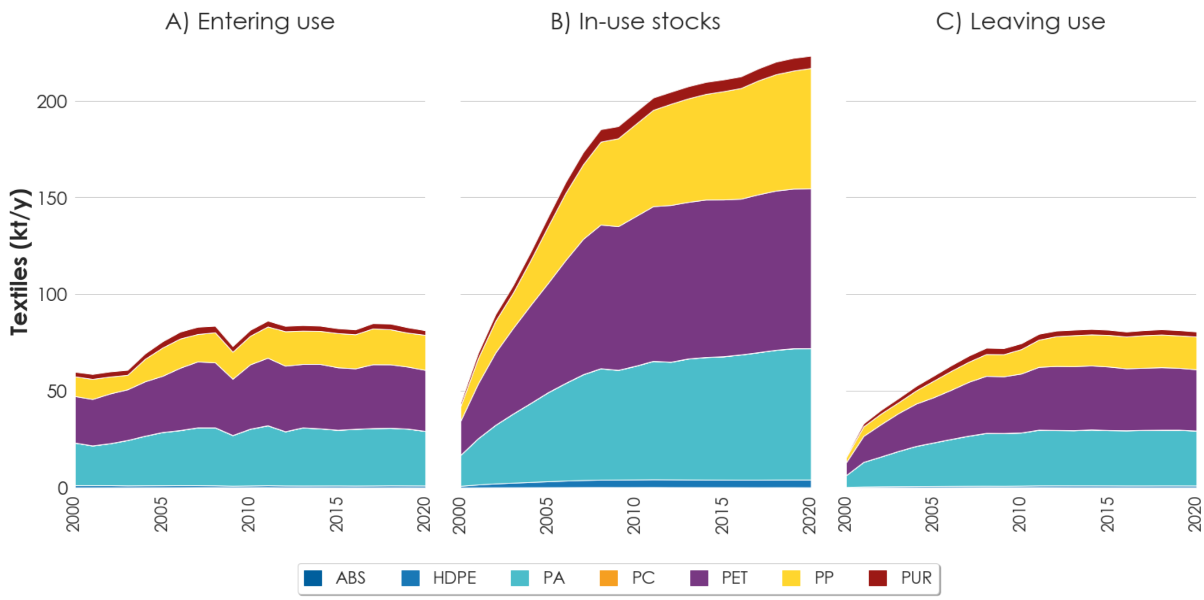

Figure S14. Annual plastics A) entering use, B) in-use stocks, and C) leaving use, distinguished for the polymer type between 2000 and 2020 for the textiles sector.

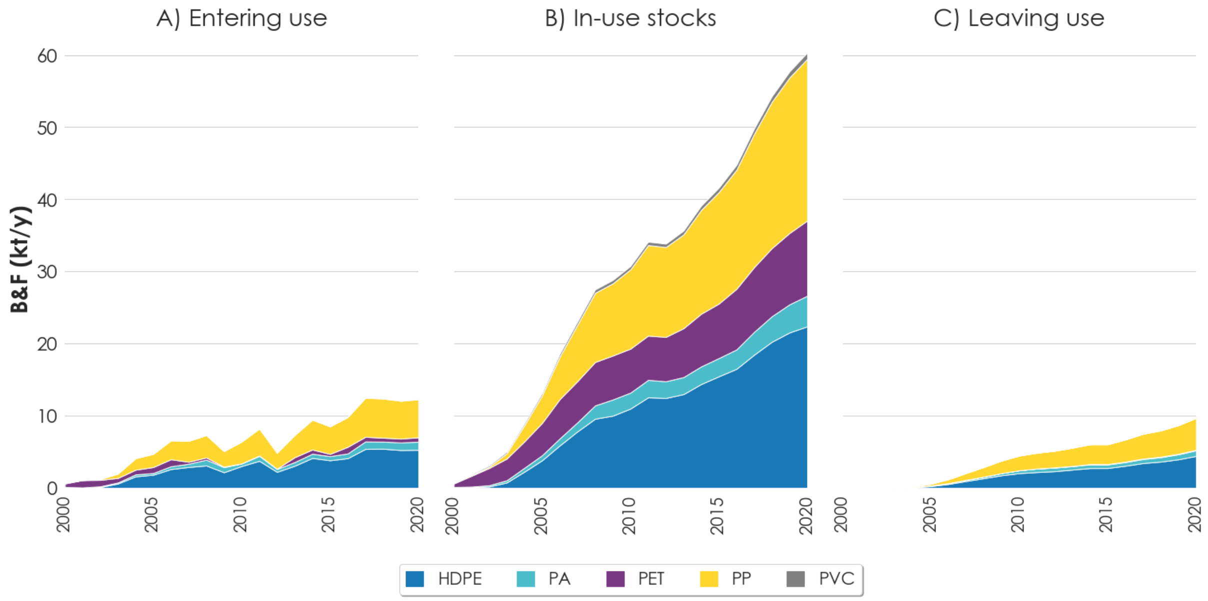

Figure S15. Annual plastics A) entering use, B) in-use stocks, and C) leaving use, distinguished for the polymer type between 2000 and 2020 for the boats and fisheries (B&F) sector.

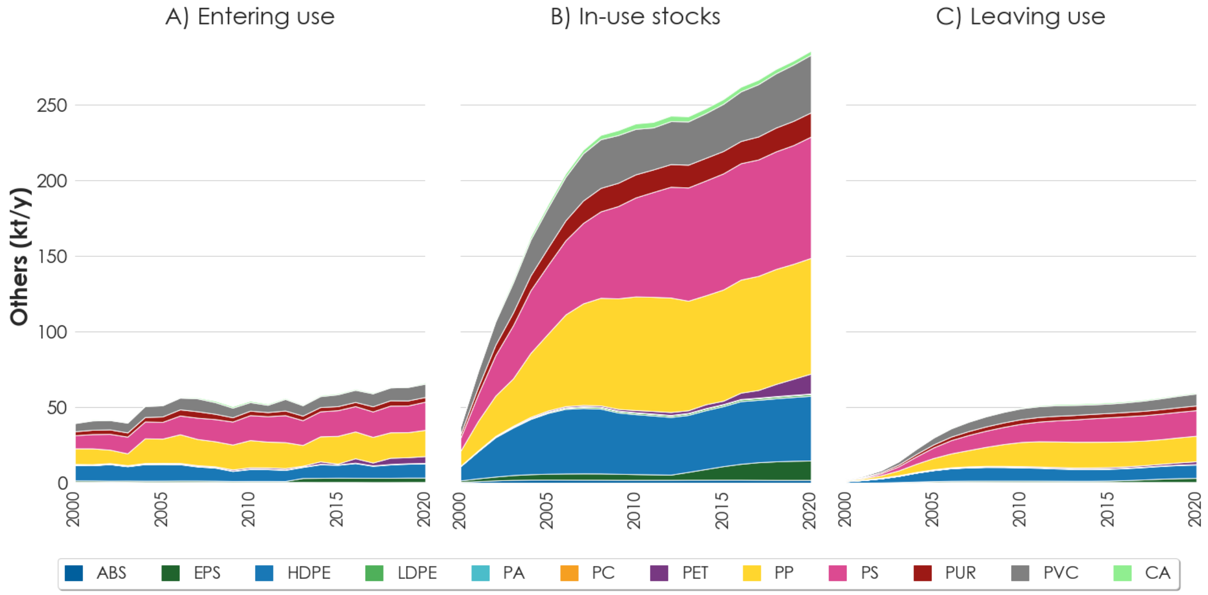

Figure S16. Annual plastics A) entering use, B) in-use stocks, and C) leaving use, distinguished for the polymer type between 2000 and 2020 for other plastics sector.

S9.3 Absolute inflow amounts to sinks and emission factors per individual product category

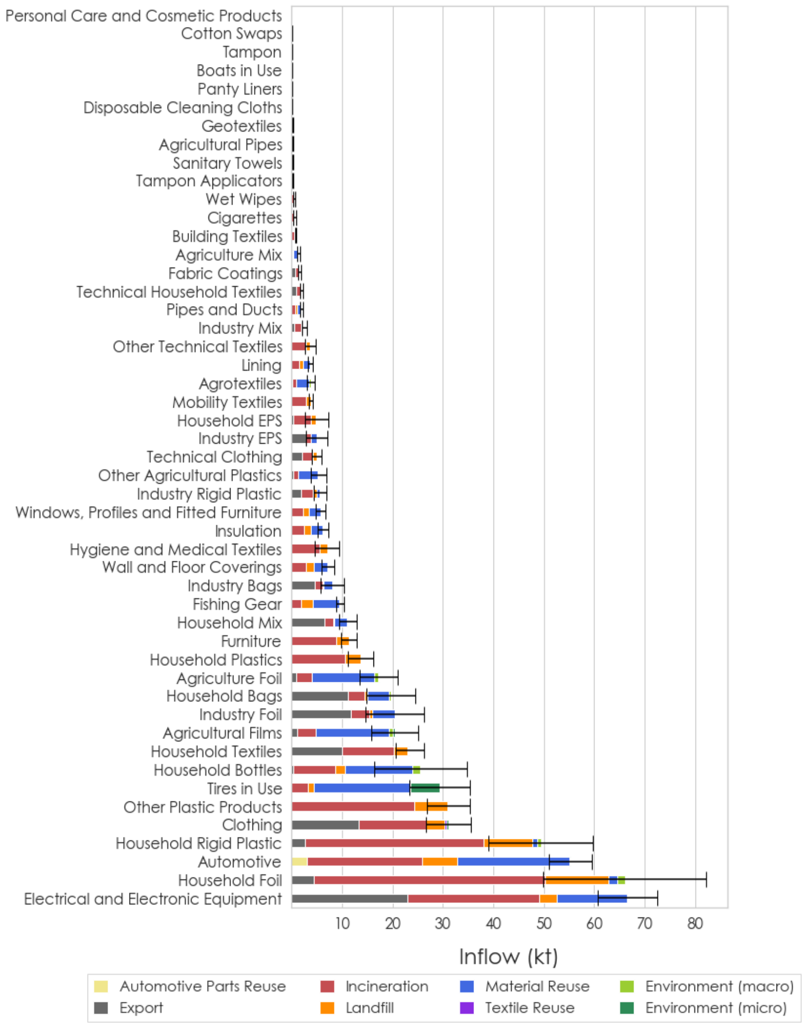

Figure S17. Absolute inflow amounts (kt) of individual product categories to all sinks in 2020.

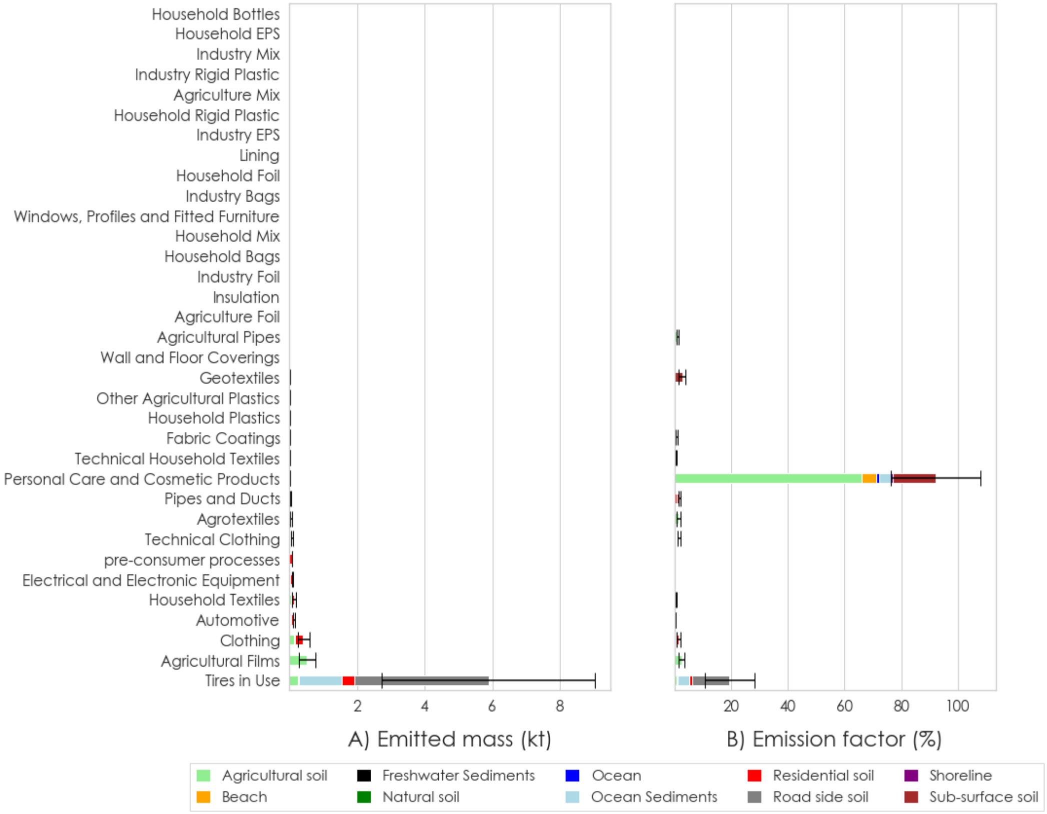

Figure S18. A) Absolute emitted mass (kt) of microplastics in 2020 for individual product categories to the environmental sinks and B) emission factors (%). Emissions factors calculated as the total inflow into each environmental sink divided by the total outflow leaving the stocks for each product category.

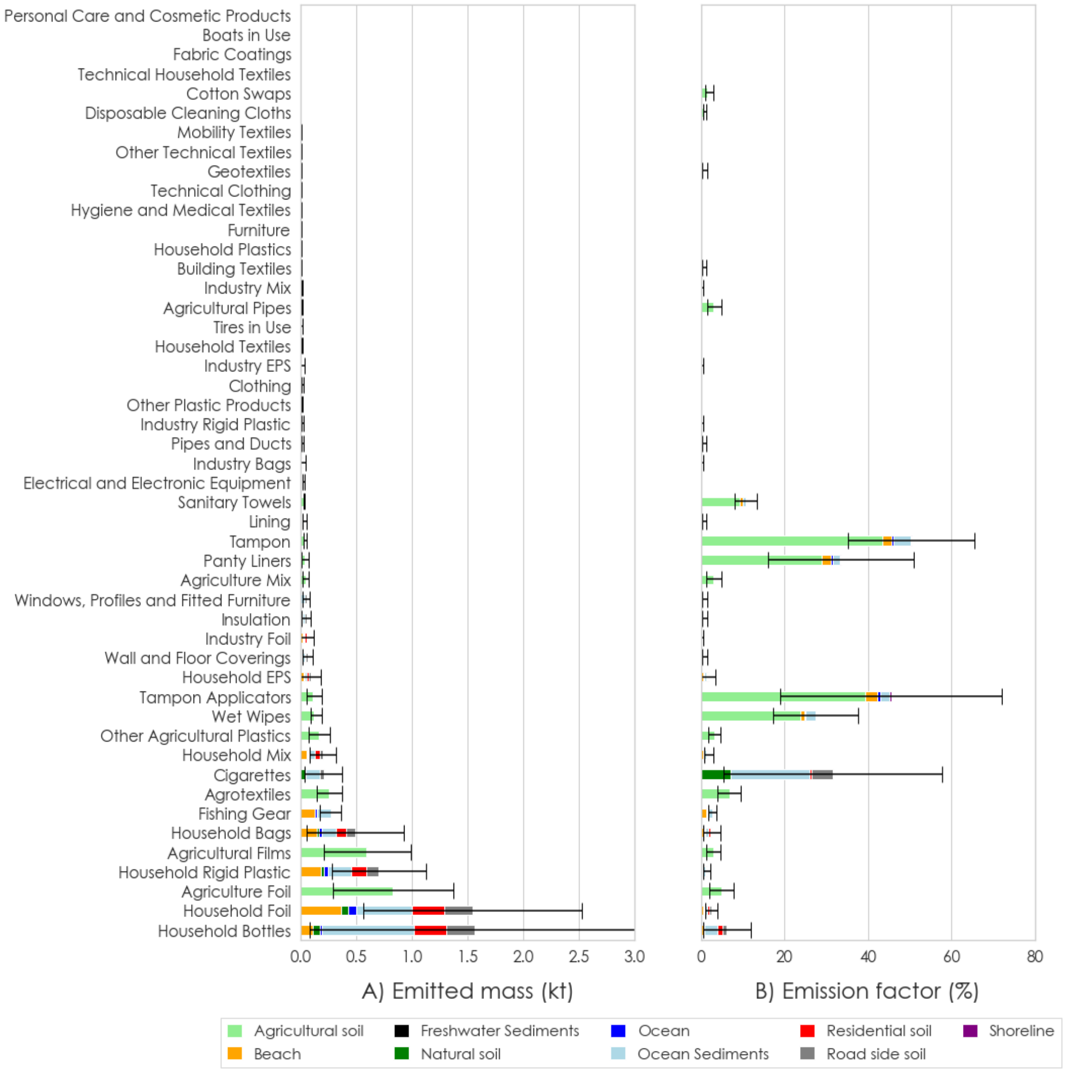

Figure S19. A) Absolute emitted mass (kt) of macroplastics in 2020 for individual product categories to the environmental sinks and B) emission factors (%). Emissions factors calculated as the total inflow into each environmental sink divided by the total outflow leaving the stocks for each product category.

S9.4 Cumulative and annual inflows into the environmental compartments

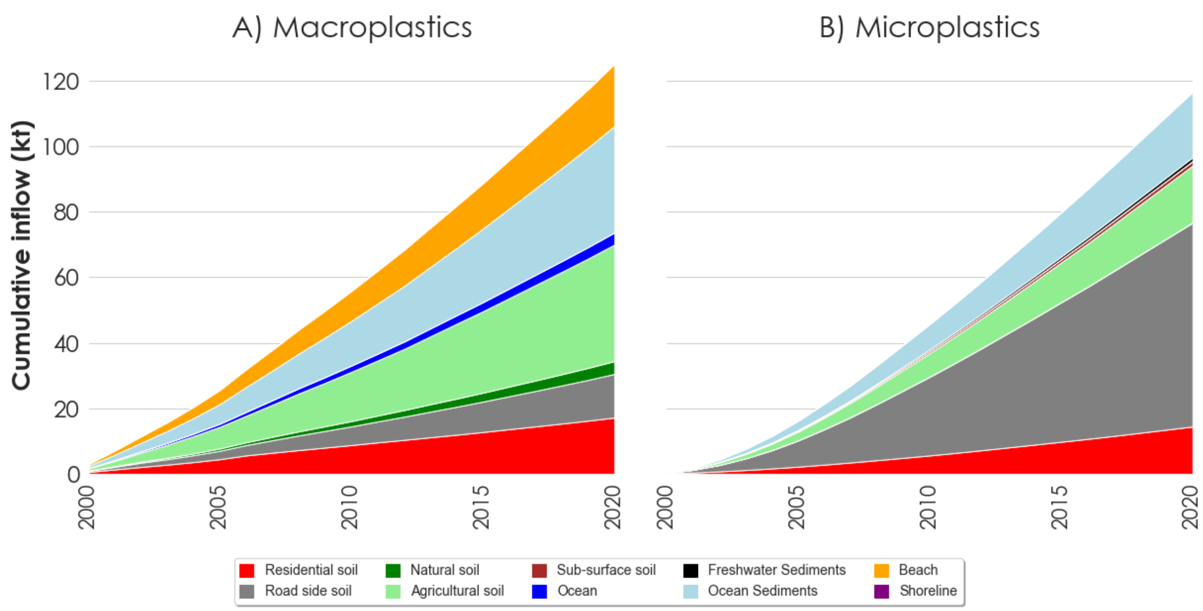

Figure S20. Cumulative plastic release of A) macroplastics and B) microplastics per receiving environmental compartment between 2000 and 2020.

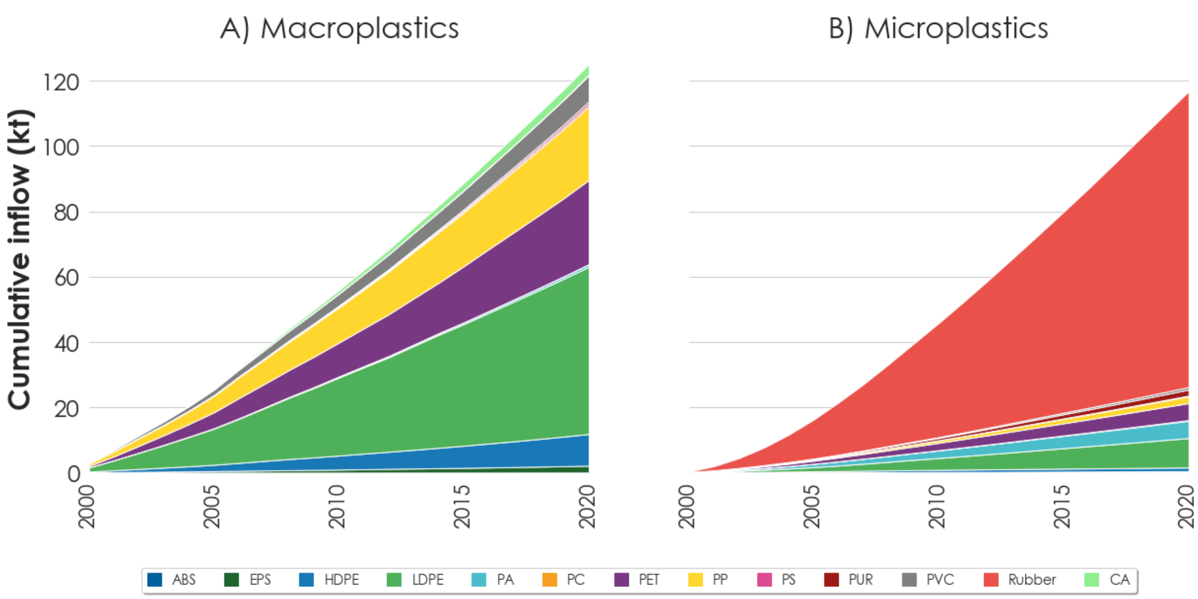

Figure S21. Cumulative plastic release of A) macroplastics and B) microplastics per polymer type between 2000 and 2020.

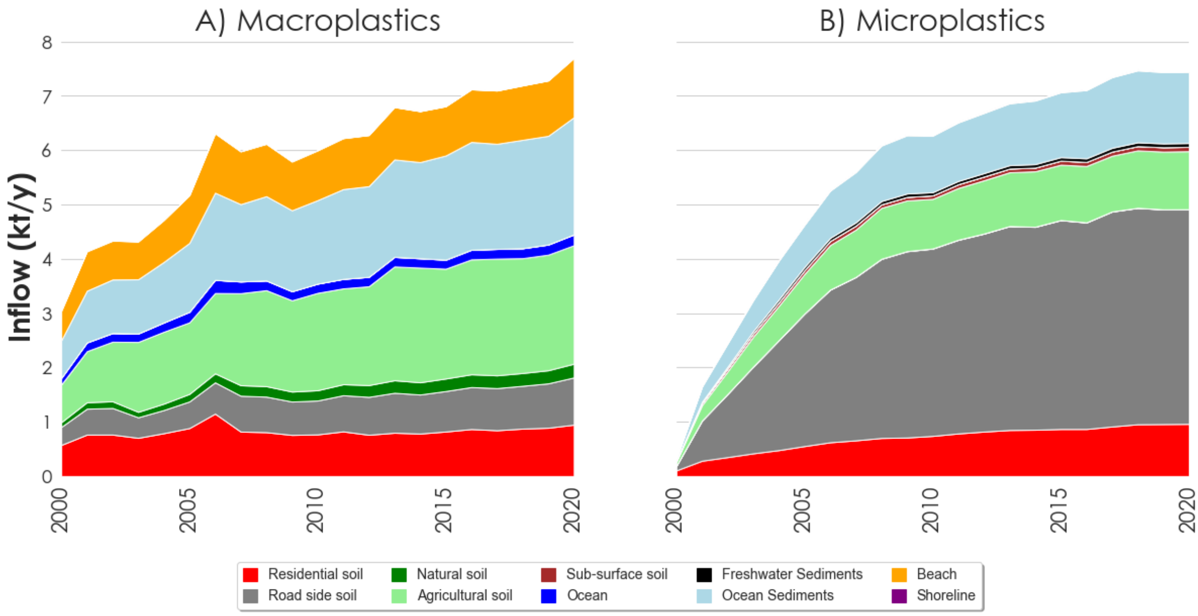

Figure S22. Annual plastic release of A) macroplastics and B) microplastics per receiving environmental compartment between 2000 and 2020.

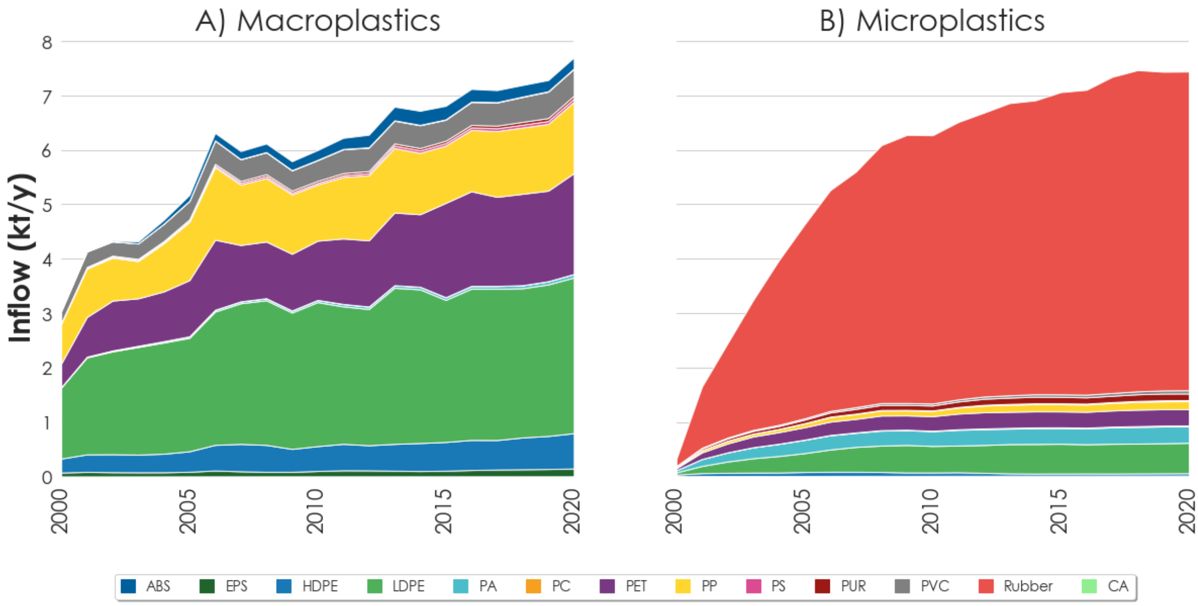

Figure S23. Annual plastic release of A) macroplastics and B) microplastics per polymer type between 2000 and 2020.

S9.5 Additives

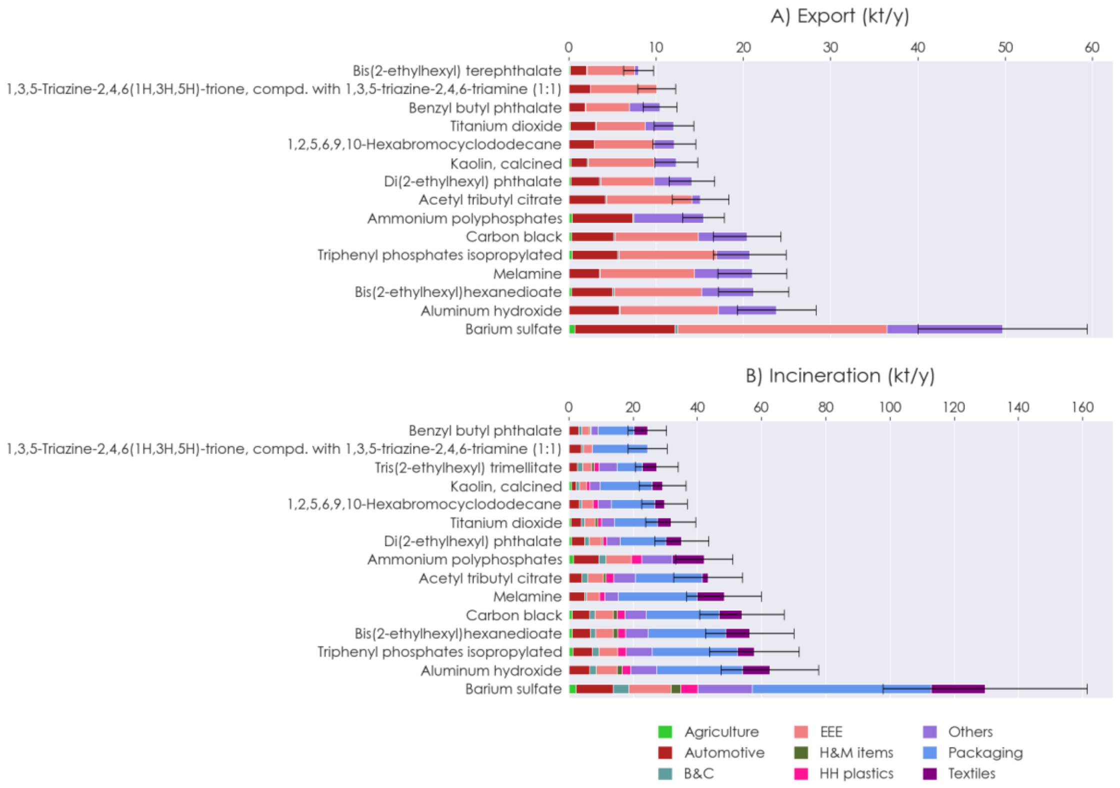

Figure S24. Maximum potential inflow amounts of the top 15 plastic additives per application sector in 2020 to A) Export, and B) Incineration. B&C: Building and Construction; EEE: Electrical and Electronic Equipment; HH Plastics: Household plastics; H&M items: Hygiene and medical items.

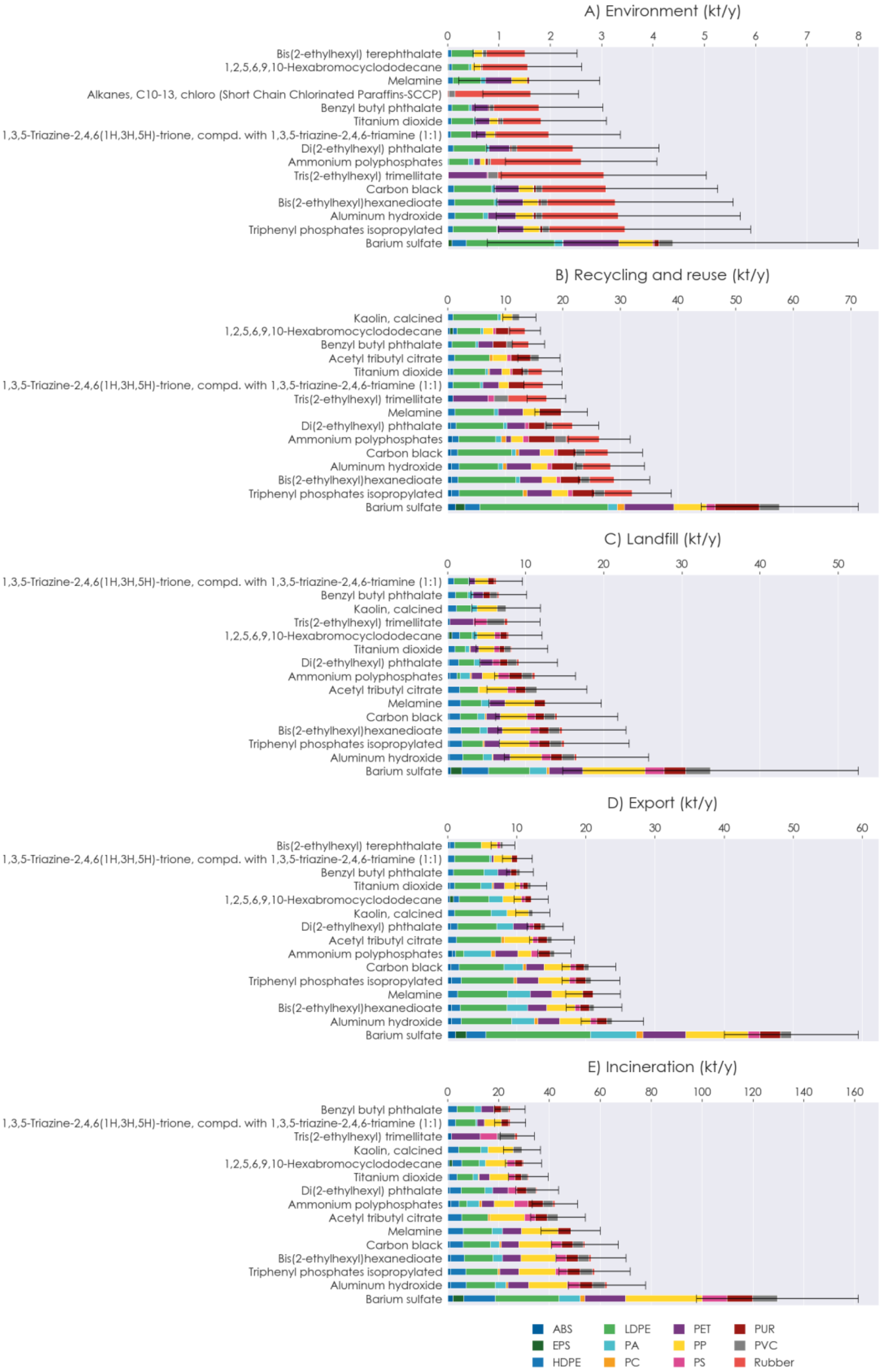

Figure S25. Maximum potential inflow amounts of the top 15 plastic additives per polymer type in 2020 to A) Environmental sinks, B) Recycling and reuse sinks, C) Landfill, D) Export, and E) Incineration.

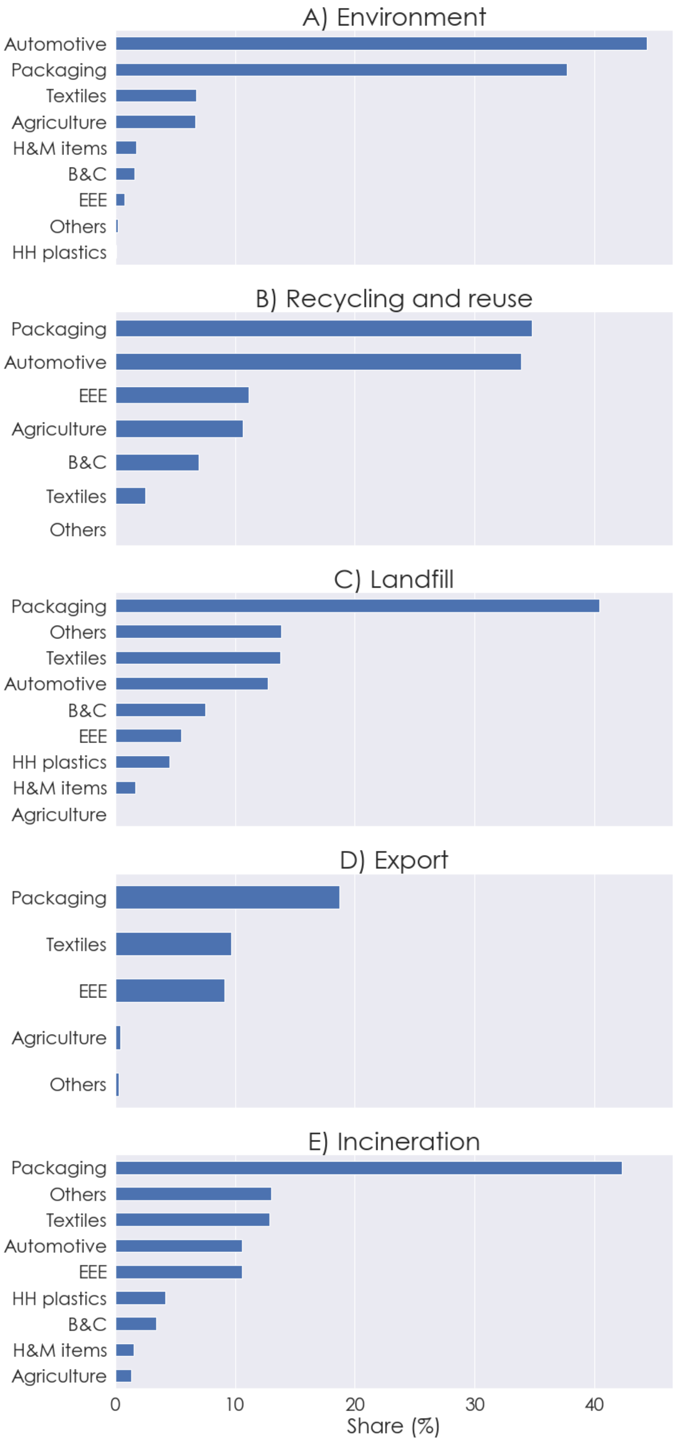

Figure S26. Source of total additive amounts to A) Environment, B) Recycling and reuse, C) Landfill, D) Export, and E) Incineration.

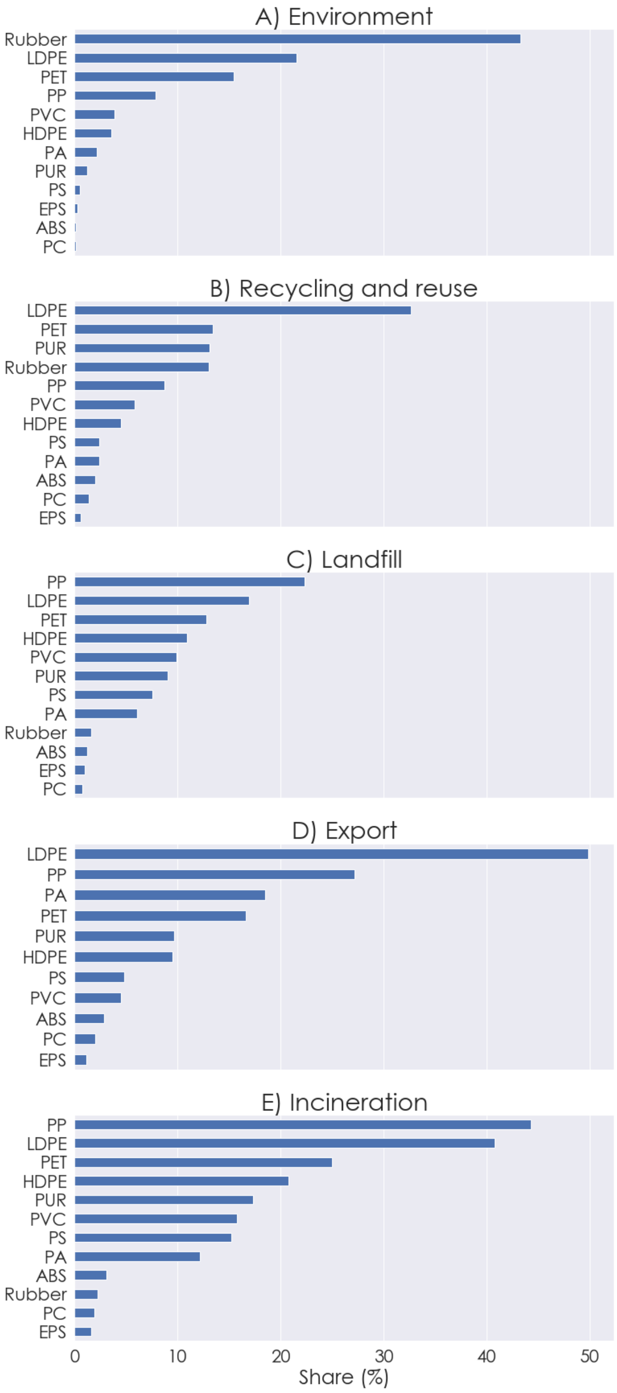

Figure S27. Polymer types associated with the total additive amounts to A) Environment, B) Recycling and reuse, C) Landfill, D) Export, and E) Incineration.

S9.6 Comparison to previous studies

Table S15. Comparison of plastic flows in the anthroposphere using the results of this study and previous studies.

Process	Study	Geographical scope	Temporal scope	Value	This study	Difference
Entering use	Kawecki et al.[35]	Europe	2016	90 kg/cap	109 ± 29 kg/capita*	21%
	Kawecki et al.[29]	Switzerland	2014	86 ± 20 kg/cap	104 ± 28 kg/capita*	21%
	Abbasi et al.[1]	Norway	2020	620 ± 23 kt	758 kt ± 197***	22%
	Systemiq[36]	Norway	2021	710 kt		7%
In-use stocks	Kawecki et al.[35]	Europe	2016	465 kg/capita	538 ± 44 kg/capita****	16%
	Abbasi et al.[1]	Norway	2020	3400 ± 56 kt	4467 ± 330 kt***	31%
	Syversen et al.[37]	Norway	2020	3109 kt		43%
Leaving use	Abbasi et al.[1]	Norway	2020	460 ± 20 kt	589 ± 113 kt****	28%
	Syversen et al.[37]	Norway	2020	540 kt		9%
	Systemiq[36]	Norway	2021	502 kt		17%

*Obtained using LDPE, HDPE, PP, PS, EPS, PET, PVC and the Norwegian population.[38]

**Derived using a total loss of 706 ± 161 kt and a population size of 8.19 million in 2014 in Switzerland.[39]

****Equivalent to the outflows released from the stocks and excluding pre-consumer wastes.

****Obtained using all 13 polymer types from this study.

Table S16. Comparison of plastic released to the environment using the results of this study and previous studies.

Study	Geographical scope	Temporal scope	Processes covered	Polymers	Receiving environmental compartment	Plastic size	Derived value	This study (Norway; equivalent parameters)	Differences
Kawecki and Nowack[4]	Switzerland	2014	Total	LDPE, HDPE, PP, PS, EPS, PET, PVC	Total	Macro	553 ± 144.9 g/cap/a	1244 ± 812 g/cap/a	1. Static MFA. 2. Land-locked country. 3. Microplastics modeled as lifetime outflows
						Micro	74.8 ± 15.1 g/cap/a	212 ± 90.0 g/cap/a	
Liu and Nowack[2]	Switzerland	2018	Total	PUR, ABS, PA, PC	Total	Macro and micro	57.7 ± 24.2 g/cap/a*	108 ± 52 g/cap/a	1. Static MFA 2. Land-locked country 3. Microplastics modeled as lifetime outflows
Luan et al.[40]	China	2020	Total	LDPE, HDPE, PP, PS, PET, PVC, ABS	Ocean	Macro	2.6 Mt (1842.7 g/cap/a**)	3.2 ± 2 kt (590 ± 400 g/cap/a)	Combined stock-driven and a release model
						Micro	196.8 kt (139.5 g/cap/a**)	39 ± 24 t (7 ± 5 g/cap/a)	
					Soil	Macro	10.2 Mt	4 ± 3 kt	

							(7228.9 g/cap/a**)	(750 ± 580 g/cap/a)	
						Micro	155.1 kt (109.9 g/cap/a**)	1.1 ± 0.5 t (204 ± 85 g/cap/a)	
Sieber et al.[5]	Switzerland	2018	Total	Rubber	Total	Micro	960 ± 350 g/cap/a	1115 ± 600 g/cap/a	Microplastics modeled as lifetime outflows
Schwarz et al.[24]	Country-specific dataset (Norway)	2020	Total	HDPE, LDPE, PET, PP, PS, EPS, PVC, rubber	Soil	Macro	20.58 kt	4.1 ± 3 kt	1. Overestimated results<br>2. No coverage for land-based sources for flows to the marine environment<br>3. No specific release pathways for coastal regions
						Micro	4.36 kt	5.6 ± 3 kt	
					Freshwater	Macro	19.7 kt	2.5 ± 0.002 t	
						Micro	745 t	66 ± 0.1 t	
					Ocean (only from fishing activities)	Macro	29.1 kt	268 ± 140 t	
Jambeck et al.[13]	Country-specific dataset (Norway)	2010	Total	Plastic	Ocean	Macro	1.26 -3.36 kt	2.6 ± 1.7 kt	Missing polymer and product types
Deshpande et al.[12]	Norway	2016	Commercial fishing gear	PE, PP, PA	Ocean	Macro	380 ± 100 t	170 ± 85 t	1. Static MFA<br>2. Aggregation of product categories and generalization of loss rates in our study
Sundt et al.[41]	Norway	2020	Plastic pellets	Plastic	Total	Micro	170 t	75 ± 11 t	1. Different modeling approach<br>2. Missing polymer and product types
			Synthetic consumer textiles	Plastic	Total	Micro	1017 t	640 ± 250 t	
			tires	Plastic	Total	Micro	8.3 t	5.9 ± 3.2 kt	
			Personal consumer products	Plastic	Total	Micro	92 t	11 ± 3 t	
			Agricultural plastics	Plastic	Total		150 t	580 ± 275 t	

*Derived using a total loss of 491 ± 206 t and a population size of 8.51 million in 2018 in Switzerland.[39]

**Derived using a population size of 1.411 billion in 2020 in China.[42]

Table S17. Comparison of additives emissions using the results of this study and previous studies.

Additive	Study	Geographical scope	Temporal scope	Value	This study (2020)	Difference
Di(2-ethylhexyl) phthalate	Cui et al.[43]	China	2020	570.7 kt (404 g/cap*)	2.4 ± 1.6 kt (454 ± 300 g/cap)	1. Release quantified using emission factors. 2. No release through plastic leakage
	COWI[44]	Europe	2007	11.6 kt (26.5 g/cap)		
	Muchangos et al.[45]	Japan	2020	32.5 kt (257 g/cap**)		
Hexabromocyclododecane	Morf et al.[46]	Switzerland	2020	64 kg (0.0074 g/capita***)	1.56 ± 1 kt (291 ± 190 g/cap)	

*Derived using a population size of 1.411 billion in 2020 in China.[42]

**Derived using a population size of 126.26 million in 2020 in Japan.[42]

***Derived using a population size of 8.63 million in 2020 in Switzerland.[39]

S9.7 Relative uncertainty

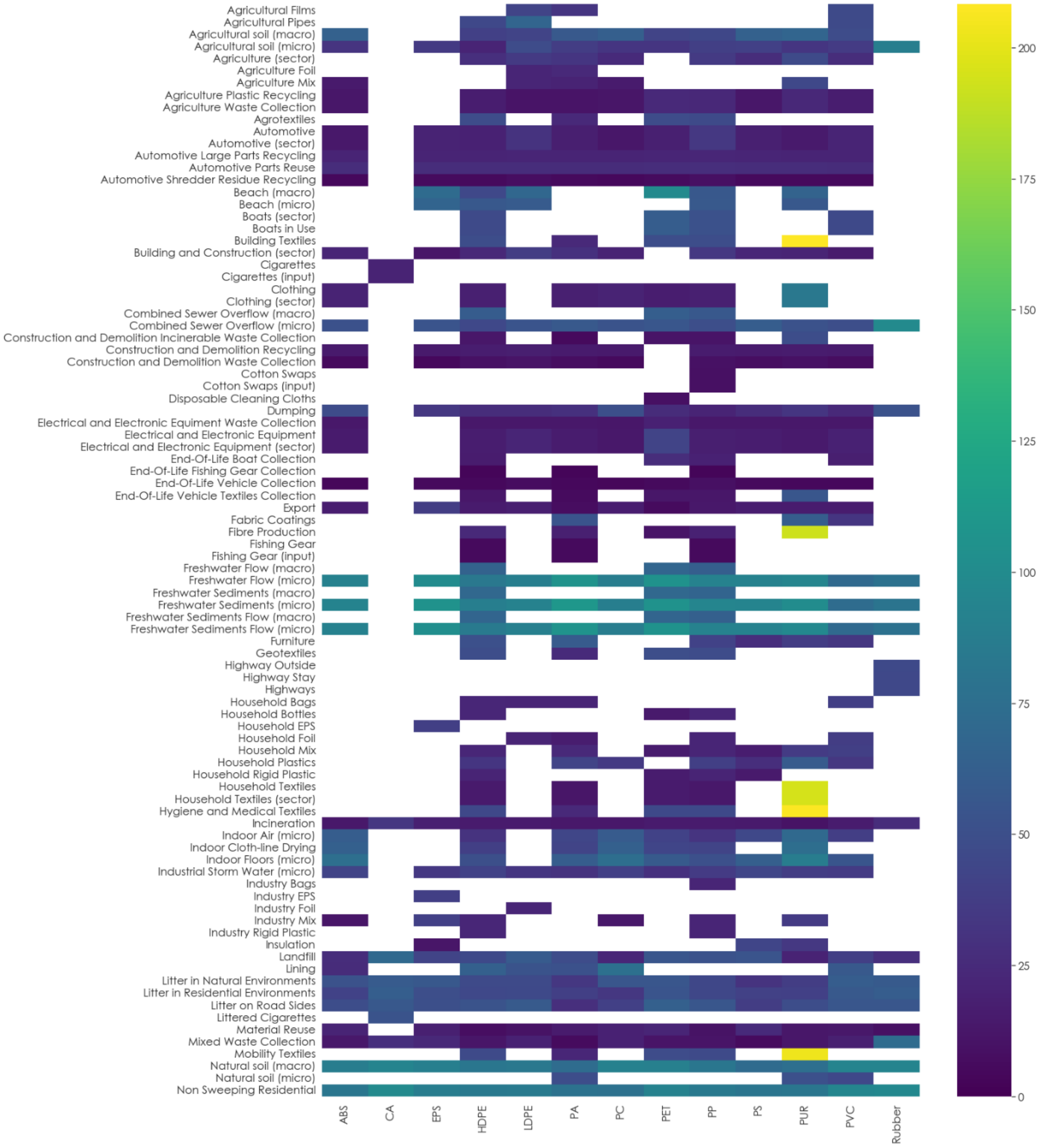

Figure S28. Relative uncertainty for the inflows per compartment (standard deviation/mean) for all polymers in 2020 (part 1).

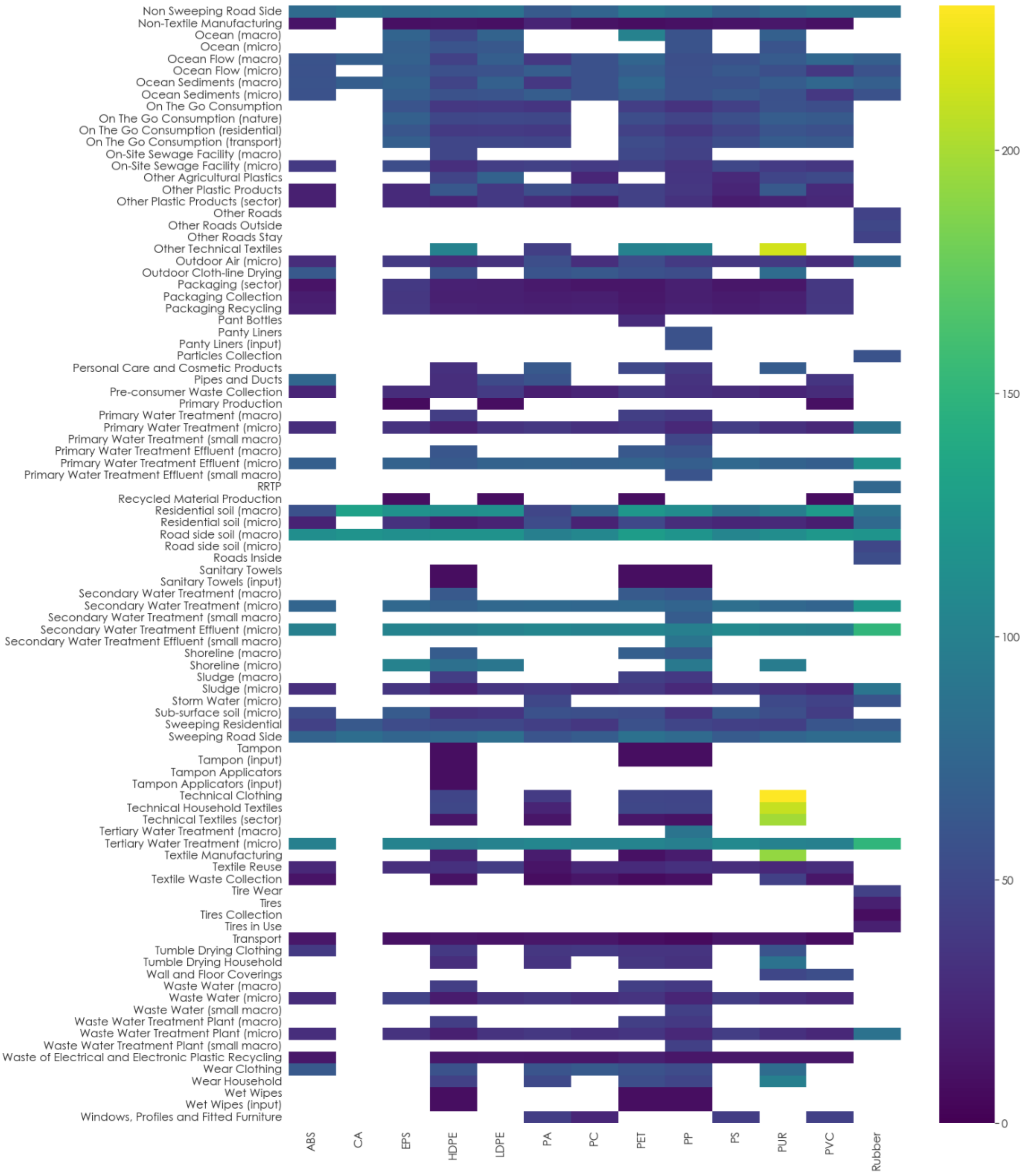

Figure S29. Relative uncertainty for the inflows per compartment (standard deviation/mean) for all polymers in 2020 (part 2).

S10 References

(1) Abbasi, G.; Hauser, M.; Baldé, C. P.; Bouman, E. A. A High-Resolution Dynamic Probabilistic Material Flow Analysis of Seven Plastic Polymers; A Case Study of Norway. *Environ. Int.* **2023**, *172*, 107693. https://doi.org/10.1016/j.envint.2022.107693.

(2) Liu, Z.; Nowack, B. Probabilistic Material Flow Analysis and Emissions Modeling for Five Commodity Plastics (PUR, ABS, PA, PC, and PMMA) as Macroplastics and Microplastics. *Resour. Conserv. Recycl.* **2022**, *179*, 106071. https://doi.org/10.1016/j.resconrec.2021.106071.

(3) Statistics Norway. *Household waste, by region, material, treatment, downstream system, contents and year*. https://www.ssb.no/en/statbank/table/13136/ (accessed 2023-04-11).

(4) Kawecki, D.; Nowack, B. Polymer-Specific Modeling of the Environmental Emissions of Seven Commodity Plastics As Macro- and Microplastics. *Environ. Sci. Technol.* **2019**, *53* (16), 9664–9676. https://doi.org/10.1021/acs.est.9b02900.

(5) Sieber, R.; Kawecki, D.; Nowack, B. Dynamic Probabilistic Material Flow Analysis of Rubber Release from Tires into the Environment. *Environ. Pollut.* **2020**, *258*, 113573. https://doi.org/10.1016/j.envpol.2019.113573.

(6) Trondheim Kommune. *Kommunal Plan for Avfall Og Avfallsreduksjon*; 2007. https://www.trondheim.kommune.no/globalassets/10-bilder-og-filer/11-politikk-og-planer/planer/temaplaner/kommunal-plan-for-avfall-og-avfallsreduksjon.pdf (accessed 2023-04-11).

(7) Geodata Online. *Demografi Admin*. https://dokumentasjon.geodataonline.no/docs/Temakart/Demografi - Admin/ (accessed 2023-08-22).

(8) OECD. *GDP and spending - Gross domestic product (GDP)*. http://data.oecd.org/gdp/gross-domestic-product-gdp.htm (accessed 2023-04-11).

(9) Friedler, E.; Brown, D. M.; Butler, D. A Study of WC Derived Sewer Solids. *Water Sci. Technol.* **1996**, *33* (9), 17–24. https://doi.org/10.1016/0273-1223(96)00365-4.

(10) Spence, K. J.; Digman, C.; Balmforth, D.; Houldsworth, J.; Saul, A.; Meadowcroft, J. Gross Solids from Combined Sewers in Dry Weather and Storms, Elucidating Production, Storage and Social Factors. *Urban Water J.* **2016**, *13* (8), 773–789. https://doi.org/10.1080/1573062X.2015.1025081.

(11) Norwegian Environment Agency. *Reduced Littering of Single-Use Plastics*; 2019. https://www.miljodirektoratet.no/publikasjoner/2019/mai-2019/reduced-littering-of-single-use-plastics/ (accessed 2023-03-10).

(12) Deshpande, P. C.; Philis, G.; Brattebø, H.; Fet, A. M. Using Material Flow Analysis (MFA) to Generate the Evidence on Plastic Waste Management from Commercial Fishing Gears in Norway. *Resour. Conserv. Recycl. X* **2020**, *5*, 100024. https://doi.org/10.1016/j.rcrx.2019.100024.

(13) Jambeck, J. R.; Geyer, R.; Wilcox, C.; Siegler, T. R.; Perryman, M.; Andrady, A.; Narayan, R.; Law, K. L. Plastic Waste Inputs from Land into the Ocean. *Science* **2015**, *347* (6223), 768–771. https://doi.org/10.1126/science.1260352.

(14) Meijer, L. J. J.; van Emmerik, T.; van der Ent, R.; Schmidt, C.; Lebreton, L. More than 1000 Rivers Account for 80% of Global Riverine Plastic Emissions into the Ocean. *Sci. Adv.* **2021**, *7* (18), eaaz5803. https://doi.org/10.1126/sciadv.aaz5803.

(15) Ellen Macarther Foundation. *A New Textiles Economy: Redesigning Fashion’s Future*; 2017. https://ellenmacarthurfoundation.org/a-new-textiles-economy (accessed 2023-05-10).

(16) Levi Strauss & Co. -; 2015. http://levistrauss.com/wp-content/uploads/2015/03/Full-LCA-Results-Deck-FINAL.pdf (accessed 2023-05-10).

(17) Lassen, C.; Hansen, S. F.; Magnusson, K.; Hartmann, N. B.; Rehne Jensen, P.; Nielsen, T. G.; Brinch, A. *Microplastics: Occurrence, Effects and Sources of Releases to the Environment in Denmark*; Report; Danish Environmental Protection Agency: Copenhagen K, 2015.

(18) Peano, L.; Kounina, A.; Magaud, V.; Chalumeau, S.; Zgola, M.; Boucher, J. *The Plastic Leak Project Guidelines*; 2020. https://quantis-evo-2022.whostaging.fr/report/the-plastic-leak-project-guidelines/ (accessed 2022-06-28).
(19) Grigoratos, T.; Martini, G. *Non-Exhaust Traffic Related Emissions. Brake and Tyre Wear PM*; 2014. https://doi.org/10.2790/22000.
(20) Wik, A.; Dave, G. Occurrence and Effects of Tire Wear Particles in the Environment – A Critical Review and an Initial Risk Assessment. *Environ. Pollut.* **2009**, *157* (1), 1–11. https://doi.org/10.1016/j.envpol.2008.09.028.
(21) Hillenbrand, T.; Dominik, T.; Boehm, E. *Einträge von Kupfer, Zink und Blei in Gewässer und Böden - Analyse der Emissionspfade und möglicher Emissionsminderungsmaßnahmen*; Umweltbundesamt, 2005. https://www.umweltbundesamt.de/publikationen/eintraege-von-kupfer-zink-blei-in-gewaesser-boeden (accessed 2023-05-23).
(22) Statistics Norway. *Municipal Wastewater - Expenditures, Investments, Wastewater Fees, Discharges, Treatment and Disposal of Sewage Sludge 2020*; 2021. https://www.ssb.no/en/natur-og-miljo/vann-og-avlop/artikler/municipal-wastewater-expenditures-investments-wastewater-fees-discharges-treatment-and-disposal-of-sewage-sludge-2020 (accessed 2022-06-27).
(23) Statistics Norway. *Land use and land cover*. Statistics Norway. https://www.ssb.no/en/natur-og-miljo/areal/statistikk/arealbruk-og-arealressurser (accessed 2023-04-09).
(24) Schwarz, A. E.; Lensen, S. M. C.; Langeveld, E.; Parker, L. A.; Urbanus, J. H. Plastics in the Global Environment Assessed through Material Flow Analysis, Degradation and Environmental Transportation. *Sci. Total Environ.* **2023**, *875*, 162644. https://doi.org/10.1016/j.scitotenv.2023.162644.
(25) van der Mheen, M.; van Sebille, E.; Pattiaratchi, C. Beaching Patterns of Plastic Debris along the Indian Ocean Rim. *Ocean Sci.* **2020**, *16* (5), 1317–1336. https://doi.org/10.5194/os-16-1317-2020.
(26) Kaandorp, M. L. A.; Dijkstra, H. A.; van Sebille, E. Closing the Mediterranean Marine Floating Plastic Mass Budget: Inverse Modeling of Sources and Sinks. *Environ. Sci. Technol.* **2020**, *54* (19), 11980–11989. https://doi.org/10.1021/acs.est.0c01984.
(27) Hurley, R.; Woodward, J.; Rothwell, J. J. Microplastic Contamination of River Beds Significantly Reduced by Catchment-Wide Flooding. *Nat. Geosci.* **2018**, *11* (4), 251–257. https://doi.org/10.1038/s41561-018-0080-1.
(28) Hurley, R. R.; Nizzetto, L. Fate and Occurrence of Micro(Nano)Plastics in Soils: Knowledge Gaps and Possible Risks. *Curr. Opin. Environ. Sci. Health* **2018**, *1*, 6–11. https://doi.org/10.1016/j.coesh.2017.10.006.
(29) Kawecki, D.; Scheeder, P. R. W.; Nowack, B. Probabilistic Material Flow Analysis of Seven Commodity Plastics in Europe. *Environ. Sci. Technol.* **2018**, *52* (17), 9874–9888. https://doi.org/10.1021/acs.est.8b01513.
(30) Statistics Norway. *Import and export - all countries and commodity numbers*. https://www.ssb.no/en/utenriksokonomi/utenrikshandel/artikler/import-og-eksport-alle-land-og-varenummer (accessed 2023-02-22).
(31) Klotz, M.; Haupt, M. A High-Resolution Dataset on the Plastic Material Flows in Switzerland. *Data Brief* **2022**, *41*, 108001. https://doi.org/10.1016/j.dib.2022.108001.
(32) PlasticsEurope. *Plastics - the Facts 2020*; Plastics Europe, 2020. https://plasticseurope.org/knowledge-hub/plastics-the-facts-2020/ (accessed 2022-06-30).
(33) EMPA. *Material- Und Energieressourcen Sowie Umweltauswirkungen Der Baulichen Infrastruktur Der Schweiz*; 2016. https://www.empa.ch/documents/56122/728861/MatCH_Bericht_Bau_v8_161017.pdf/3a733b91-ab69-43cd-ad81-2b6817716eff (accessed 2023-05-22).
(34) Wagner, M.; Scherer, C.; Alvarez-Muñoz, D.; Brennholt, N.; Bourrain, X.; Buchinger, S.; Fries, E.; Grosbois, C.; Klasmeier, J.; Marti, T.; Rodriguez-Mozaz, S.; Urbatzka, R.; Vethaak, A. D.; Winther-Nielsen, M.; Reifferscheid, G. Microplastics in Freshwater Ecosystems: What We Know and What

We Need to Know. *Environ. Sci. Eur.* **2014**, *26* (1), 12. https://doi.org/10.1186/s12302-014-0012-7.
(35) Kawecki, D.; Wu, Q.; Gonçalves, J. S. V.; Nowack, B. Polymer-Specific Dynamic Probabilistic Material Flow Analysis of Seven Polymers in Europe from 1950 to 2016. *Resour. Conserv. Recycl.* **2021**, *173*, 105733. https://doi.org/10.1016/j.resconrec.2021.105733.
(36) Systemiq. *Achieving Circularity: A Low-Emissions, Circular Plastic Economy in Norway*; 2023. https://www.systemiq.earth/reports/achieving-circularity/ (accessed 2023-08-04).
(37) Syversen, F.; Sundt, P.; Kirkevaag, K.; Briedis, R. *Materialstrømmen til plast i Norge – hva vet vi?*; Mepex Consult AS, 2020. https://mepex.no/fagbibliotek/materialstrommen-til-plast-i-norge-hva-vet-vi/ (accessed 2022-06-27).
(38) Statistics Norway. *Population, by sex and one-year age groups (M) 1986 - 2023.* https://www.ssb.no/en/statbank/table/07459 (accessed 2023-10-11).
(39) Eurostat. *Population change - Demographic balance and crude rates at national level*. https://ec.europa.eu/eurostat/databrowser/view/DEMO_GIND/default/table?lang=en (accessed 2023-02-27).
(40) Luan, X.; Kou, X.; Zhang, L.; Chen, L.; Liu, W.; Cui, Z. Estimation and Prediction of Plastic Losses to the Environment in China from 1950 to 2050. *Resour. Conserv. Recycl.* **2022**, *184*, 106386. https://doi.org/10.1016/j.resconrec.2022.106386.
(41) Sundt, P.; Haugedal, S. R.; Rem, T.; Schulze, P.-E. *Norske landbaserte kilder til mikroplast - Norwegian land-based sources of microplastics*; Miljødirektoratet, 2020. https://www.miljodirektoratet.no/publikasjoner/2021/april-2021/norske-landbaserte-kilder-til-mikroplast/ (accessed 2022-08-12).
(42) The World Bank. *World Development Indicators*. https://databank.worldbank.org/reports.aspx?source=2&series=SP.POP.TOTL&country=CHN (accessed 2023-07-18).
(43) Cui, Y.; Chen, J.; Wang, Z.; Wang, J.; Allen, D. T. Coupled Dynamic Material Flow, Multimedia Environmental Model, and Ecological Risk Analysis for Chemical Management: A Di(2-Ethylhexhyl) Phthalate Case in China. *Environ. Sci. Technol.* **2022**, *56* (15), 11006–11016. https://doi.org/10.1021/acs.est.2c03497.
(44) *Data on Manufacture, Import, Export, Uses and Releases of Bis (2-Ethylhexyl) Phthalate (DEHP) as Well as Information on Potential Alternatives to Its Use*; COWI A/S: Denmark, 2009. https://echa.europa.eu/documents/10162/8fd5a74b-6807-42b6-ae1f-d1d7f04f40f8 (accessed 2023-12-27).
(45) Muchangos, L. dos; Xue, M.; Zhou, L.; Kojima, N.; Machimura, T.; Tokai, A. Flows, Stocks, and Emissions of DEHP Products in Japan. *Sci. Total Environ.* **2019**, *650*, 1007–1018. https://doi.org/10.1016/j.scitotenv.2018.09.077.
(46) Morf, L. S.; Buser, A. M.; Taverna, R.; Bader, H.-P.; Scheidegger, R. Dynamic Substance Flow Analysis as a Valuable Risk Evaluation Tool – A Case Study for Brominated Flame Retardants as an Example of Potential Endocrine Disrupters. *CHIMIA* **2008**, *62* (5), 424–424. https://doi.org/10.2533/chimia.2008.424.
